# CRISPR/Cas9 and iPSC-Based Therapeutic Approaches in Alzheimer’s Disease

**DOI:** 10.3390/antiox14070781

**Published:** 2025-06-25

**Authors:** Ivana Raffaele, Giovanni Luca Cipriano, Ivan Anchesi, Salvatore Oddo, Serena Silvestro

**Affiliations:** 1 IRCCS Centro Neurolesi “Bonino-Pulejo”, Via Provinciale Palermo, Contrada Casazza, 98124 Messina, Italy; ivana.raffaele@irccsme.it (I.R.); giovanniluca.cipriano@irccsme.it (G.L.C.); ivan.anchesi@irccsme.it (I.A.); 2 Department of Chemical, Biological, Pharmaceutical and Environmental Sciences, University of Messina, Viale F. Stagno d’Alcontres 31, 98166 Messina, Italy; salvatore.oddo@unime.it

**Keywords:** Alzheimer’s disease, CRISPR/Cas9, genome editing, stem cell, aging, neuroinflammation, oxidative stress

## Abstract

Alzheimer’s disease (AD), the leading cause of dementia, remains poorly understood despite decades of intensive research, which continues to hinder the development of effective treatments. As a complex multifactorial disorder, AD lacks a cure to halt the progressive neurodegeneration, and the precise mechanisms underlying its onset and progression remain elusive, limiting therapeutic options. Due to the challenges of studying neuronal cells in vivo, technologies such as clustered regularly interspaced short palindromic repeats/CRISPR-associated protein 9 (CRISPR/Cas9) and human-induced pluripotent stem cells (hiPSCs) are key for identifying therapeutic targets, although they face technical and ethical hurdles in their early stages. CRISPR/Cas9 and hiPSCs are promising for disease modeling and therapy, but off-target effects and the complexity of gene editing in the brain limit their use. CRISPR technology enables specific genetic modifications in key AD-related genes, such as *APP*, *PSEN1*, *PSEN2*, and *APOE*, providing valuable insights into disease mechanisms. iPSC-derived neurons, astrocytes, microglia, and 3D organoids can recapitulate key aspects of human AD pathology, but they do not fully replicate the complexity of the human brain, limiting clinical applicability. These technologies advance studies of amyloid processing, tau aggregation, neuroinflammation, and oxidative stress, yet translating them into clinical therapies remains challenging. Despite the promise of CRISPR/Cas9 and iPSCs for precision medicine, gaps in knowledge about their long-term safety and efficacy must be addressed before clinical implementation.

## 1. Introduction

Alzheimer’s disease (AD) is a chronic, degenerative condition that significantly affects brain function and represents the leading cause of dementia worldwide, particularly among women aged over 65 years [1]. Typically, progressive neuronal damage causes cognitive decline, affecting memory, language, orientation, and behavior, primarily due to irreversible brain atrophy [2]. As the prevalence of AD continues to rise, there is an urgent need to develop effective treatments or preventive strategies. Currently, no curative treatment exists, and the available options offer only limited symptomatic relief [1].

The main pathological hallmarks of AD include the accumulation of beta-amyloid (Aβ) peptides and the abnormal phosphorylation of Tau protein. These alterations lead to extracellular plaques and intracellular neurofibrillary tangles in the brain, respectively. These phenomena are typically associated with neurodegeneration [3,4,5].

Beyond the accumulation of Aβ and Tau, additional processes such as neurodegeneration and synaptic degeneration are linked to disease progression [6,7,8]. Chronic glial activation can exacerbate neuronal loss, while plaques and tangles can disrupt communication, impairing neuronal plasticity. However, the molecular basis of these processes is still not fully understood [9]. Among these molecular processes, oxidative stress has emerged as a critical contributor to AD pathogenesis, as it exacerbates neuronal injury through the accumulation of reactive oxygen species and disruption of mitochondrial function [10].

The complex nature of AD, with diverse genetic, molecular, and clinical manifestations, complicates the development of a unified model [11]. In most cases, late-onset AD is genetically and etiologically heterogeneous, with innumerable genes and environmental factors involved in disease progression. In contrast, familial AD (FAD) is rare (<1%) and results from autosomal dominant mutations in genes such as amyloid precursor protein (APP), presenilin 1 (PSEN1), and presenilin 2 (PSEN2) [12].

A key obstacle in AD research concerning therapeutic strategies arises from the difficulties associated with in vivo models, which often fail to replicate human neuropathology [13,14] accurately. Genotype–environment interactions and differences in experimental approaches further prevent animal models from simulating human disease, limiting the translational impact of preclinical findings [11,15]. Although some therapies have shown promise, their clinical efficacy remains modest, and side effects pose concerns. Thus, innovative strategies are needed to better understand the molecular mechanisms underlying AD and to identify new, more effective interventions [16].

In this context, combining genetic and cellular therapies has emerged as a promising direction, slowing disease progression and possibly restoring specific cellular functions [17]. As explored in Section 2, Clustered Regularly Interspaced Short Palindromic Repeats/CRISPR-associated protein 9 (CRISPR/Cas9) has revolutionized gene editing approaches in AD by enabling targeted manipulation of pathogenic loci [18]. Preclinical studies in animal models of AD reported that gene therapy could lower Aβ deposition and enhance cognitive function, alongside a reduction in inflammation and neurodegeneration [19]. At the same time, stem cell-based approaches have gained attention for their potential in neurodegeneration. Several stem cell types, including neural stem cells (NSCs), mesenchymal stem cells (MSCs), embryonic stem cells (ESCs), and human-induced pluripotent stem cells (hiPSCs), have been studied for their ability to differentiate into functional neuronal and glial cells [17]. Moreover, three-dimensional (3D) brain organoids derived from hiPSCs have shown the capacity to replicate key AD processes. They are used to test the efficacy of drugs despite limitations in mimicking complex brain interactions, cell maturation, and aging [20]. CRISPR, combined with hiPSCs, enables precise introduction or correction of genetic variants, such as single nucleotide polymorphisms (SNPs), without altering the entire genome. This approach provides a powerful platform to investigate the functional consequences of specific genetic alterations, enhancing the accuracy of disease modeling, drug screening, and gene function analysis [20]. Despite its potential, the limitations of the model must be considered. Challenges include off-target effects, delivery inefficiency, and mosaicism for CRISPR, as well as developmental immaturity, genomic instability, and cellular heterogeneity in neurons and iPSC-derived organoids. These technical and biological constraints reduce the fidelity of the model and limit translational applicability.

This review aims to provide a comprehensive and critical view of the opportunities offered by integrated therapies to overcome the limitations of current therapies, improving the management and treatment of AD.

## 2. CRISPR-Based Gene Editing in AD: Target Genes, Therapeutic Potential, and Challenges

CRISPR/Cas9 is a powerful gene-editing tool with growing potential to study and treat neurodegenerative diseases such as AD, which currently lack effective therapies [21,22,23,24]. CRISPR/Cas9 has been applied to identify and edit genes involved in oxidative stress, a key contributor to AD pathogenesis, offering potential therapeutic avenues to mitigate related neuronal damage [25,26]. Since most mutations in the APP, PSEN1, and PSEN2 genes are associated with increased Aβ production, the use of CRISPR/Cas9 to correct these mutations in brain cells could help reduce Aβ accumulation [27].

This, in turn, may mitigate downstream pathological processes such as oxidative stress and other hallmarks of aging and neurodegenerative diseases.

### 2.1. Mechanism of CRISPR/Cas9

**Figure 1:**
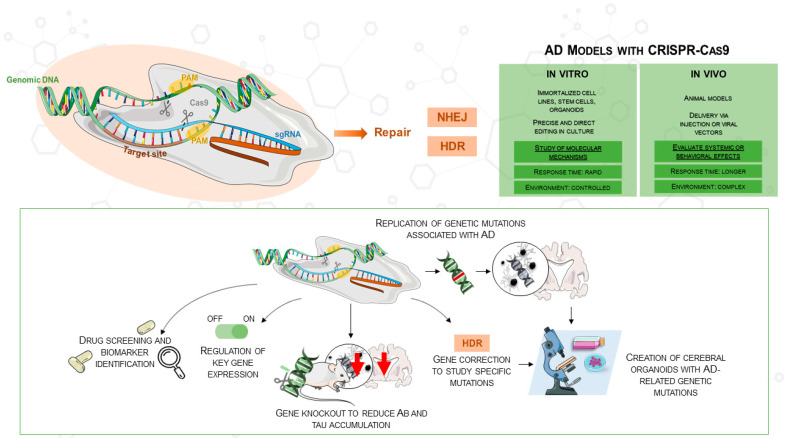
The figure illustrates the use of CRISPR/Cas9 in AD research, highlighting its application in both in vitro and in vivo models for gene knockouts, mutations, and gene correction. CRISPR/Cas9 enables the replication of AD mutations, the regulation of gene expression, and the creation of cerebral organoids from hiPSCs. The comparison table outlines in vitro (e.g., cell lines, organoids) and in vivo (e.g., animal models) models in AD research, depicting CRISPR/Cas9’s role in advancing therapeutic development. This image was created using the image bank of Servier Medical Art (Available online: http://smart.servier.com/; accessed on 30 May 2025) licensed under a Creative Commons Attribution 3.0 Unported License (available online: https://creativecommons.org/licenses/by/3.0/, accessed on 30 May 2025). AD: Alzheimer’s disease; CRISPR/Cas9: Clustered Regularly Interspaced Short Palindromic Repeats/CRISPR-associated protein 9; DSB: double-strand breaks; sgRNA: single guide RNA; NHEJ: non-homologous end joining; HDR: homology-directed repair; Aβ: amyloid beta.

Various neurodegenerative diseases have been investigated with well-established methods such as TALENs and zinc-finger nucleases. However, CRISPR/Cas9 has become a more easily available and flexible method, especially fit for AD research [28]. Originally discovered in the bacterial adaptive immune system, CRISPR/Cas9 targets DNA sequences with the Cas9 nuclease in concert with a guide RNA (gRNA). A double-stranded break (DSB) is produced when the nuclease binds to its target, activating intrinsic repair mechanisms that might produce either homology-directed repair (HDR) or non-homologous end joining (NHEJ). This technique can be used to introduce or fix mutations connected to diseases. CRISPR/Cas9 consists of two main components: a single-guide RNA (sgRNA) that guides Cas9 to the matching target DNA sequence and a Cas9 protein that cleaves the target DNA. Usually 5′-NGG-3′, a brief protospacer adjacent motif (PAM), is necessary immediately downstream of the target for Cas9 recognition. Reprogramming CRISPR/Cas9 usually requires the design of a new sgRNA rather than changing a protein scaffold; thus, this RNA-based targeting mechanism offers many more advantages than gene-editing techniques dependent on engineered protein–DNA interactions [28]. Moreover, since CRISPR/Cas9 allows the combination of multiple sgRNAs in a single experiment, multiplex gene editing is enhanced, enabling the simultaneous targeting of several genes involved in Alzheimer’s disease (Figure 1). This approach not only accelerates research but also facilitates the creation of more accurate disease models, as shown in the comparison of in vitro and in vivo applications in the figure.

### 2.2. Gene-Editing Potential of CRISPR/Cas9 in AD Models

CRISPR/Cas9 technology has emerged as a versatile and promising tool for investigating and manipulating key genetic mechanisms involved in AD pathogenesis [29,30]. Precise genetic manipulation holds promise for improving patient outcomes [31]. CRISPR/Cas9 enables the creation of genetically modified models (in vivo and in vitro) to target key AD mechanisms, such as Aβ plaque deposition, Tau hyperphosphorylation, and neuroinflammation. For further details, refer to Table 1.

Kwart et al. used CRISPR/Cas9 to alter Aβ production in mice with mutant APP, reducing toxic Aβ peptides without impairing brain function. In a separate set of experiments, they also targeted other genes implicated in AD pathology. Specifically, they used CRISPR/Cas9 to downregulate BACE1, a key enzyme involved in Aβ generation, and to modulate genes involved in neuroinflammation. Additionally, CRISPR-based editing increased the expression of neurotrophic factors such as brain-derived neurotrophic factors (BDNF), contributing to enhanced neuronal protection and synaptic repair. Overall, these genetic modifications not only improved synaptic plasticity but also led to significant cognitive improvements in behavioral tests [32]. In addition, by reducing Aβ accumulation, these genetic corrections may also alleviate oxidative stress, which is known to contribute to neurodegeneration in AD [33]. The connection between Aβ and oxidative stress has been well established: Aβ peptides can bind redox-active metal ions such as copper and iron, which catalyze the formation of reactive oxygen species (ROS), contributing to cellular damage [34]. Several molecular mechanisms have been described in which oxidative stress and the two hallmark proteins, Aβ and tau, could be associated, creating a vicious cycle that accelerates disease progression, impairing neural and cognitive function [35]. It is likely that Aβ-dependent oxidative damage may be caused by the inhibition of mitochondrial oxidative phosphorylation by Aβ, accelerating neurodegeneration and highlighting the role of ROS in Aβ toxicity [36].

Aβ production begins with the cleavage of APP by the enzyme β-secretase, which releases C-terminal membrane amino acid fragments. The membrane-bound C-terminal fragment is further degraded by γ-secretase to produce the Aβ1-40 and Aβ1-42 isoforms [37]. The Aβ1-42 isoform contributes to amyloid plaque formation due to its propensity for self-aggregation. Aβ1-42 production is linked to mutations in *APP*, *PSEN1*, and *PSEN2* genes, with APOE4 increasing Aβ accumulation and AD risk [38,39]. CRISPR/Cas9 has been used to manipulate PSEN1 and PSEN2, key components of γ-secretase involved in Aβ production, by creating knockout cell lines in mouse neuroblastoma cells (N2A). These models have been used to study genetic variants commonly found in hereditary AD, providing insight into Aβ production and oxidative stress [40]. However, the effects of PSEN1 mutations on γ-secretase activity are complex. While some mutations increase the Aβ42/Aβ40 ratio and contribute to disease, others may reduce Aβ production or cause an imbalance in unexpected ways. Sun et al. (2017) demonstrated that certain PSEN1 mutations alter γ-secretase activity, with varying effects on Aβ production, highlighting the need to consider mutation-specific variability when exploring γ-secretase modulation in AD therapies [41]. In another study, the *PSEN1^M146L^* allele, known to promote Aβ42 generation and cause early-onset AD, was selectively ablated in human fibroblasts using CRISPR/Cas9. The technology successfully mutated more than 50% of the alleles harboring the mutation across samples from multiple families, resulting in a decreased Aβ42/40 ratio in the culture medium. Thus, the use of CRISPR/Cas9 by selectively targeting the *PSEN1^M146L^* allele could have the ability to partially restore the Aβ42/40 ratio imbalance and counteract the AD-associated phenotype [42]. A similar result was obtained in another study in which CRISPR/Cas9 technology was used to correct the *PSEN2^N141I^* mutation associated with autosomal dominant early-onset FAD [43]. Another target for the CRISPR/Cas9 system is mutations in the APP gene. CRISPR/Cas9 was applied to selectively eliminate the *APPswe* (*Swedish*) allele both ex vivo and in vivo, reducing Aβ levels [44]. However, while this approach shows promise, the long-term effects and off-target risks of such genetic modifications require further evaluation. In contrast, another study utilized CRISPR/Cas9 technology to introduce a new mutation (E674K) in the *APP* gene. The A673T mutation was successfully introduced in 53% of HEK293T cells, resulting in reduced Aβ levels [45]. However, the long-term safety and potential off-target effects of such mutations remain major concerns that require further validation. While these findings suggest that CRISPR/Cas9 could be a valuable therapeutic tool for AD patients with *APPswe* and other point mutations increasing Aβ levels, the risks of off-target effects and the need for rigorous validation before clinical application are significant concerns.

In another study, CRISPR/Cas9 was used to induce *SORL1* deficiency in a preclinical AD model in pigs. Knockout of the *SORL1* gene slowed endosomal recycling, indirectly increasing the levels of Aβ and tau in cerebrospinal fluid. These results highlight the importance of CRISPR/Cas9 in studying early disease stages and testing preventive treatments [28]. The use of this technology can be extended to identify new mutations. In fact, in one study, a mutation (p.T209S) was induced in the ZDHHC21 gene, associated with a Chinese family affected by FAD. The mutation in this gene increased the palmitoylation of key proteins, such as APP and FYN, contributing to the accumulation of Aβ and phosphorylation of tau. In addition, mutant mice showed significant cognitive decline, as well as reduced synaptic plasticity and neuronal loss, effects related to the hyperpalmitoylation of FYN and the activation of the NMDAR2B receptor, causing neuronal excitotoxicity. Using CRISPR/Cas9 to alter post-translational modifications like palmitoylation, researchers identified a previously unexplored mechanism in AD pathogenesis. Moreover, the use of palmitoylation inhibitors like 2-BP improved synaptic plasticity and reduced neurodegeneration, suggesting a potential therapeutic strategy for AD patients [46].

The link between Aβ and oxidative stress is well established: Aβ binds redox-active metals, such as copper and iron, facilitating the production of ROS that contribute to neuronal damage. CRISPR/Cas9 interventions targeting *APP*, *PSEN1/2*, and *BACE1* have been shown to reduce ROS generation and inflammation, thereby improving synaptic plasticity and neuronal viability [47]. Beyond amyloid pathology, CRISPR/Cas9 has also been employed to modulate neuroinflammatory responses, a central feature of AD progression. Activated microglia, in response to Aβ and tau, can produce pro-inflammatory cytokines that exacerbate neuronal injury. Gene editing strategies targeting *TREM2*, a key modulator of microglial activation, have yielded mixed outcomes [48,49].

In a study using TauPS2APP (which develop both amyloid and tau pathology) and P301L homozygous (which develop only tau pathology without Aβ) mouse models, researchers examined early and late disease stages, monitoring tau accumulation, brain atrophy, and microglial response over the lifespan of the mice (from 9 to 17 months). Deletion of the TREM2 gene using gene editing worsened tau accumulation and accelerated brain atrophy, especially in the presence of Aβ. These results indicate the potential of CRISPR/Cas9 to intervene in TREM2, regulate the microglial response, reduce inflammation, and slow AD progression [50]. In compliance with these data, in another study, *TREM2* knockouts showed abnormal microglial activation and altered inflammatory responses, particularly in a pro-inflammatory context stimulated with peripheral lipopolysaccharide (LPS) [51]. In a recent study, researchers introduced the Y38C variant into the Trem2 gene, showing how its mutation leads to impaired synaptic function and altered microglial responses, even in early stages and in the absence of pathological triggers such as Aβ [52]. These findings underline the importance of gene editing in modulating inflammatory processes and microglial responses in AD models.

Another study has further explored the effect of gene editing on neuroinflammation and synaptic plasticity. CRISPR/Cas9 was used to delete the *CysLT1R* gene in an APP/PS1 mouse model of AD, which showed Aβ accumulation and cognitive decline. Knocking out this gene significantly reduced the accumulation of neurotoxic and unstable Aβ species in the brain, leading to a decrease in plaque formation and associated pathological changes. This reduction reduces the levels of pro-inflammatory cytokines such as TNF-α and IL-6, which modulate the microglial response, thus reducing the immune response. Indeed, gene modification reduced NF-κB activation, a key transcription factor in inflammation, decreasing amyloidosis and enhancing synaptic protection. Thus, *CysLT1R* knockout mice showed improved synaptic plasticity and cognitive performance and increased long-term potentiation [53].

**Figure 2:**
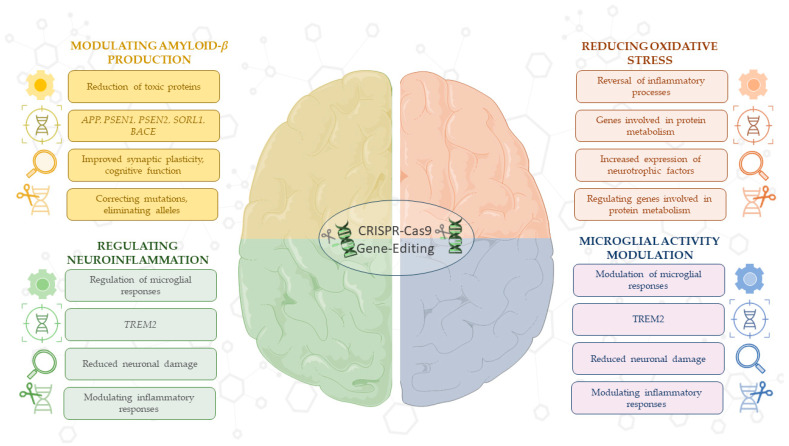
This schematic illustrates the therapeutic versatility of CRISPR/Cas9 in targeting key processes in AD. CRISPR/Cas9 enables the regulation of Aβ production through gene editing in critical AD-related genes, such as *APP, PSEN1*, *PSEN2*, *SORL1,* and *BACE*, while also enhancing synaptic function. Technology modulates neuroinflammation and microglia activity, particularly through genes like TREM2, to reduce neuronal damage. Additionally, CRISPR/Cas9 reduces oxidative stress by reversing inflammatory pathways, regulating genes involved in protein metabolism, and promoting neurotrophic factor release. However, challenges remain in the efficiency of CRISPR/Cas9 delivery, including limitations in targeting specific tissues and concerns about off-target effects. These applications highlight CRISPR’s potential as a therapeutic strategy for AD, despite current delivery barriers. For detailed procedures, refer to Table 1. This image was created using the image bank of Servier Medical Art (Available online: http://smart.servier.com/; accessed on 30 May 2025) licensed under a Creative Commons Attribution 3.0 Unported License (available online: https://creativecommons.org/licenses/by/3.0/, accessed on 30 May 2025). APP: Amyloid precursor protein; PSEN1: presenilin 1; PSEN2: presenilin 2; SORL1: sortilin-related receptor 1; BACE: beta-site APP-cleaving enzyme; TREM2: triggering receptor expressed on myeloid cells 2; CRISPR/Cas9: clustered regularly interspaced short palindromic repeats/CRISPR-associated protein 9.

Overall, the results of these preclinical studies emphasize the importance of using CRISPR/Cas9 technology to modulate molecular and cellular processes, such as Aβ production and reduction of oxidative stress, underlying the pathogenesis of AD. This technology, alone or in combination with other treatments, could identify promising therapeutic strategies aimed at halting or even reversing AD progression (Figure 2).

**Table 1:** The table summarizes the main preclinical evidence for CRISPR/Cas9 gene editing in AD research. It includes studies focusing on the modulation of Aβ production and oxidative stress, as well as studies targeting neuroinflammation and microglial activity. In detail, the table outlines the objective of the studies, the model and experimental design used, together with the main results and their therapeutic implications. These studies highlight how precise gene editing can advance our understanding of AD’s pathogenesis and offer promising prospects for targeted interventions.

Study	Technology	Aim	Model	Experimental Design	Result	Implications	Ref.
CRISPR/Cas9 in mouse models to edit *APP* and *PSEN*	CRISPR/Cas9	Correct genetic mutations in APP and *PSEN1/PSEN2* genes to reduce Aβ production	Transgenic mouse models	Mouse models with correction of *APP/PSEN* mutations, measurement of Aβ reduction and cognitive improvement	Reduction in beta-amyloid plaques and improvement in cognitive function	Therapeutic potential of CRISPR/Cas9 to reduce the production of toxic proteins	[32]
Knockout of *PSEN1/PSEN2* in murine N2A cells	CRISPR/Cas9	Knockout *PSEN1/PSEN2* to study γ-secretase’s role in APP metabolism and Aβ production	Murine N2A cell lines	Creation of *PSEN1/PSEN2* knockout in N2A cells and study of genetic variants and their effects on the Aβ42/Aβ40 ratio	Elimination of Aβ production and identification of variants increasing the Aβ42/Aβ40 ratio	Platform for testing genetic mutations in the presenilin genes	[40]
CRISPR/Cas9-mediated *PSEN1* knockout in N2a cells	CRISPR/Cas9	Assess the impact of 138 *PSEN1* mutations on Aβ40 and Aβ42 production	In vitro reconstitution in N2A-*PSEN1/2KO-8/71* cells	Expression of wild-type and mutant *PSEN1*; measurement of Aβ levels	90% of mutations reduced Aβ40 and Aβ42 production, while 10% decreased the Aβ42/Aβ40 ratio	Provides insights into how *PSEN1* mutations affect γ-secretase activity, informing CRISPR-based strategies to correct or compensate for these mutations	[41]
CRISPR/Cas9 corrects the *PSEN1^M146L^* mutation in a FAD model	CRISPR/Cas9	To evaluate whether CRISPR/Cas9 can selectively disrupt the *PSEN1^M146L^* allele in human fibroblasts and normalize the Aβ 42/40 ratio, a hallmark of AD	Human fibroblasts with the *PSEN1^M146L^* mutation were transfected with CRISPR/Cas9 plasmids	In vitro use of CRISPR/Cas9 to correct the *PSEN1^M146L^* mutation, followed by analysis of Aβ production and neuronal function	Correcting the mutation restored the Aβ42/40 ratio, reducing amyloid plaque formation and improving electrophysiological properties of neurons	This study emphasizes the potential of CRISPR/Cas9 in FAD treatment by restoring normal amyloid precursor protein cleavage	[42]
CRISPR/Cas9-correctable mutation-related molecular and physiological phenotypes in iPSC-derived Alzheimer’s PSEN2^N141I^ neurons	CRISPR/Cas9	To model AD in vitro using *PSEN2^N141I^* mutant iPSC-derived BFCNs and assess whether CRISPR/Cas9 correction reverses molecular and functional phenotypes	Human iPSCs from *PSEN2^N141I^* mutation carriers and controls	PSCs were differentiated into BFCNs; Aβ42/40 levels and electrophysiological activity were measured; CRISPR/Cas9 was used to correct PSEN2N141I; functional, biochemical, and molecular assays compared mutant, control, and corrected lines	*PSEN2^N141I^* neurons showed an increased Aβ42/40 ratio and reduced spike frequency/amplitude; CRISPR/Cas9 correction normalized both molecular and physiological phenotypes	Demonstrates that *PSEN2^N141I^* contributes to AD-related dysfunctions in human neurons and that these defects are reversible through gene editing.; supports the amyloid hypothesis and use of iPSC-BFCNs for AD modeling	[43]
Human fibroblasts from *APPswe* mutation carriers and APPWT controls	CRISPR/Cas9	Disrupt the *APPswe* to reduce Aβ production in AD	Human fibroblasts from *APPswe* mutation carriers and APPWT controls	CRISPR/Cas9 was used to target the *APPswe* or *APPWT* alleles in human fibroblasts with selective gRNAs	There was a 60% reduction in secreted Aβ40 in fibroblasts. In vivo disruption of the *APPswe* allele in a mouse model led to decreased Aβ production.	It proves CRISPR/Cas9 can selectively disrupt *APP* mutations in AD models, offering a potential gene therapy for FAD	[44]
Base editing strategy for insertion of the A673T mutation in the *APP* gene to prevent AD development in vitro	CRISPR/Cas9	To insert the *A673T* mutation in the *APP* gene to reduce Aβ accumulation and prevent AD development	HEK293T and SH-SY5Y cell lines	Used Cas9 nickase-based base editors for insertion of *A673T* mutation in the 7 gene; quantification of Aβ levels and deep sequencing to evaluate editing efficiency	The A673T mutation was successfully inserted in 53% of HEK293T cells. This resulted in reduced Aβ peptide accumulation, particularly Aβ40 and Aβ42.	It suggests that base editing could be used to prevent the development of AD by modifying the *APP* gene and reducing toxic Aβ accumulation	[45]
Preclinical AD model in pigs (*SORL1* knockout)	CRISPR/Cas9	Create a preclinical AD model in pigs with *SORL1* deficiency to study the effect on Aβ and tau production	Pigs with *SORL1* knockout	Creation of *SORL1* knockout pigs to evaluate preclinical AD biomarkers, including Aβ and tau in cerebrospinal fluid	Increase in Aβ and tau in cerebrospinal fluid, without formation of amyloid plaques	Useful model for exploring the preclinical phase of AD and testing new treatments	[28]
*ZDHHC21* mutation and the role of palmitoylation	CRISPR/Cas9	Introduce the ZDHHC21 p.T209S mutation to study the effect of palmitoylation on APP and AD pathology	Mouse models with *ZDHHC21* mutation	Introduction of *ZDHHC21* mutation in mouse models to study the effect of palmitoylation on Aβ and tau	Increase in palmitoylation, Aβ production, and tau phosphorylation, leading to cognitive decline	Alternative mechanism in AD pathogenesis with therapeutic potential linked to palmitoylation	[46]
*TREM2* deletion and microglial regulation	CRISPR/Cas9	Study the role of TREM2 in regulating microglial response and tau pathology in the presence of amyloid	TauPS2 APP mice (Aβ and tau pathologies), P301Lhomo mice (tauopathy)	*TREM2* deletion in TauPS2APP mice to study the impact on microglial function and tau pathology in the presence of Aβ	*TREM2* plays a protective role in limiting tau pathology, suggesting it as a target for gene editing therapies	Worsening tau accumulation and brain atrophy in the presence of Aβ, impaired microglial function	[50]
Behavioral and transcriptomic analysis of Trem2-null mice	CRISPR/Cas9	Investigate the impact of TREM2 deficiency on neuroinflammation and microglial response in AD models	Trem2 knockout mice (VelociGene and CRISPR/Cas9 versions)	Gene editing was used to create *TREM2*-deficient mice; transcriptomic analysis and behavioral tests were performed	No significant behavioral or cognitive differences were observed in *TREM2* knockout mice; microglial activation was delayed following an LPS challenge.	The study shows that *TREM2* is crucial for modulating microglial response in neuroinflammation and suggests caution when interpreting the effects of specific gene knockout strategies in AD research	[51]
TREM2 Y38C mutation and loss of TREM2 impairs neuronal synapses in adult mice	CRISPR/Cas9	Investigate the impact of TREM2 Y38C mutation and TREM2 loss on neuronal synapse function and microglial activity in mice	Trem2 Y38C homozygous (Trem2Y38C/Y38C) and Trem2^−/−^ mice	CRISPR/Cas9 was used to generate the Trem2 Y38C mutation in the Trem2 gene of mice; mice were analyzed for synaptic protein levels, microglial morphology, and gene expression changes	TREM2 Y38C mutation impaired synaptic plasticity and myelination in hippocampal regions	The study underscores the role of TREM2 in the development of presenile dementia and highlights the importance of CRISPR/Cas9 for modeling genetic mutations linked to AD and related diseases	[52]
*CysLT1R* deletion and its effects on neuroinflammation and synaptic plasticity	CRISPR/Cas9	Evaluate the impact of *CysLT1R* deletion on amyloidosis, synaptic plasticity, cognition, and neuroinflammation	*CysLT1R* gene knockout in APP/PS1 mice to study its effects on Aβ accumulation, synaptic plasticity, and cognitive function	Reduction in toxic Aβ levels, lowered pro-inflammatory cytokines (TNF-α, IL-6), improved synaptic plasticity and cognitive performance	Another pathway through which gene editing can alleviate AD symptoms by reducing inflammation and improving synaptic protection	Reduction in toxic Aβ levels, lowered pro-inflammatory cytokines (TNF-α, IL-6), improved synaptic plasticity and cognitive performance	[53]

Aβ: Amyloid beta; AD: Alzheimer’s disease; APP: amyloid precursor protein; *APPswe*: amyloid precursor protein Swedish mutation; Cas9: CRISPR-associated protein 9; CRISPR: clustered regularly interspaced short palindromic repeats; CysLT1R: cysteinyl leukotriene receptor 1; FAD: familial AD; gRNA: guide RNA; IL-6: interleukin 6; LPS: lipopolysaccharide; N2A: neuro2A; *PSEN1*: presenilin 1; *PSEN2*: presenilin 2; SH-SY5Y: human neuroblastoma cell line; *SORL1*: sortilin-related receptor 1; TNF-α: tumor necrosis factor alpha; *TREM2*: triggering receptor expressed on myeloid cells 2; WT: wild-type; *ZDHHC21*: zinc finger DHHC-type containing 21.

### 2.3. Translating Gene Therapy into Clinical Practice for AD

While there are currently no active clinical trials using CRISPR/Cas9 for AD, gene editing technology has already been successfully tested in other neurological settings. NTLA-2001 and nexiguran ziclumeran, both CRISPR/Cas9-based therapies, showed a significant and long-lasting reduction of transthyretin protein in the serum of patients with hereditary transthyretin amyloidosis and cardiomyopathy (NCT04601051). Phase 1 results demonstrated a good safety profile, with mild and transient adverse events highlighting the feasibility of in vivo gene editing in humans [54,55].

In the field of AD and related dementias, several gene therapy trials not based on direct genome editing are currently ongoing or completed. CERE-110 (adeno-associated virus 2, AAV2- nerve growth factor, NGF, NCT00087789, NCT00876863) is an adeno-associated vector carrying the *NGF* gene. It demonstrated good tolerability in Phase 1 and 2 trials, with persistent gene expression over time, but limited vector uptake and imprecise targeting compromised its clinical efficacy. However, a long-lasting neuronal trophic response, including axonal sprouting and cellular hypertrophy, was observed [56,57].

A Phase 1 AAV-human telomerase reverse transcriptase trial (NCT04133454) explores the use of human telomerase reverse transcriptase as a strategy to extend cell life and counteract the degenerative mechanisms of AD, with potential effects on cognition and quality of life. In parallel, AAV2-BDNF (NCT05040217) aims to evaluate whether BDNF, administered continuously via viral vector, can slow neuronal loss in patients with AD or mild cognitive impairment.

In the field of frontotemporal dementia with progranulin mutations (FTD-GRN), two gene therapy approaches are currently being tested: AVB-101 (NCT06064890) and PR006 (NCT04408625). Both aim to restore progranulin levels. In mouse models, PR006 improved lysosomal and inflammatory pathological markers. Preliminary data from a Phase 1/2 trial indicate that a single intracisternal administration was generally safe and well tolerated, with increased progranulin levels in the cerebrospinal fluid in all patients. Adverse events associated with PR006 include cerebrospinal fluid pleocytosis and a transient increase in neurofilament light chain, likely associated with mild toxicity in the dorsal root ganglia [58].

Table 2 summarizes the major clinical trials of gene therapy approaches in AD and related dementias, highlighting the use of CRISPR/Cas9 and AAV vectors for therapeutic targets such as NGF, BDNF, human telomerase reverse transcriptase, and progranulin. The table provides an overview of the clinical phases, outcomes, and limitations of these trials, illustrating the therapeutic potential and challenges associated with these approaches.

## 3. Stem Cell Therapies in AD: Cell Types, Mechanisms, and Applications in Research

### 3.1. Types of Stem Cells Used in AD Therapy and Research

Stem cells represent a promising tool for AD research and therapy, owing to their regenerative potential and utility in disease modeling. Combined with genome editing tools such as CRISPR/Cas9, stem cells enable the creation of precise models to investigate AD mechanisms and personalize therapeutic strategies [59]. Their self-renewal and multipotency make stem cells fundamental in preclinical research and potential candidates for autologous transplantation strategies [60]. Stem cell therapies are under investigation for several neurodegenerative disorders [61,62,63], including AD [64], Parkinson’s disease (PD) [65], amyotrophic lateral sclerosis [66], and multiple sclerosis [67]. Based on developmental origin, stem cells are classified as embryonic, fetal, perinatal (e.g., umbilical cord-derived stem cells), and adult [68]. Based the differentiation potential, stem cells are classified as totipotent (e.g., zygote-derived stem cells) [68,69], pluripotent (e.g., ESCs, iPSCs) [70], multipotent (e.g., MSCs, NSCs), oligopotent, and unipotent, with restricted differentiation capabilities [71,72]. ESCs, derived from blastocysts, can differentiate into all cell types of the three germ layers [73,74]. In AD models, the implantation of ESC-derived cholinergic and GABAergic neurons has been shown to improve memory and promote synaptic integration. However, tumorigenic risk and ethical concerns limit clinical application [75,76]. MSCs, isolated from bone marrow, adipose tissue, or umbilical cord, are clinically attractive due to low immunogenicity and paracrine secretion of neurotrophic and anti-inflammatory factors [77]. In AD models, implantation of MSCs promotes neurogenesis, reduces Aβ burden, and modulates microglial activity [78,79,80]. NSCs differentiate into neurons, astrocytes, and oligodendrocytes [81,82]. They may support the repair of damage to blood vessels in the brain [83] and regulate immune responses [84] by replacing lost neurons, restoring neurotransmitter levels, and providing neurotrophic factors that support synaptic function and cell survival [85]. Despite being used in AD research, NSCs are not as effective as hiPSCs. NSCs are less suited for researching the intricacies of cellular interactions in AD since they are already committed to the neural lineage and have a limited ability to differentiate, mostly toward neurons and astrocytes [86,87]. NSC transplantation in AD models enhances synaptic repair and neurogenesis and reduces glial activation and pro-inflammatory markers [88,89]. The iPSCs reprogrammed from somatic cells allow patient-specific modeling of AD [90]. iPSC-derived neurons replicate pathological hallmarks, such as Aβ accumulation and tau phosphorylation, and provide platforms for drug screening and gene correction via CRISPR/Cas9.

Despite progress, major translation barriers persist. ESCs and iPSCs carry risks of uncontrolled growth and teratoma formation, influenced by reprogramming methods, transplantation site, and host background. To address this, rigorous differentiation and purification protocols are essential [91]. The immunogenicity of stem cells represents another major challenge. Even autologous iPSCs can elicit immune responses due to the expression of abnormal antigens. Developing universal stem cell lines with reduced immunogenicity may help overcome this challenge [92]. Producing clinical-grade stem cells with consistent quality and differentiation efficiency remains a major hurdle. A potential solution is the development of universal cell lines with reduced immunogenicity or the use of immunosuppressive strategies to prevent rejection in allogeneic transplants [93]. One of the main obstacles to the clinical implementation of stem cell therapies is the need for standardized protocols to produce large-scale, reproducible cell populations. Variability in differentiation methods and the quality of generated cells can impact therapeutic efficacy and treatment safety [94].

In summary, stem cells hold transformative potential in preclinical research by enabling the development of personalized and regenerative therapies. However, rigorous preclinical validation and technological advancements are essential to fully harness their capabilities and overcome the existing barriers to their clinical application (Table 3).

### 3.2. Clinical Trials on Stem Cell Therapies for AD

Stem cell therapies for AD are rapidly advancing, with several clinical trials demonstrating promising safety profiles and some early signs of efficacy. Various approaches use autologous or allogeneic MSCs from sources such as adipose tissue, umbilical cord blood, bone marrow, or perinatal tissues. Despite promising preclinical results and acceptable tolerability, stem cell therapies for AD have yet to demonstrate meaningful clinical efficacy. Advancing these approaches will require larger, well-designed studies with extended follow-up and careful consideration of translational, regulatory, and ethical challenges [95].

Autologous adipose-derived MSCs are among the most widely studied, as seen in the AstroStem trial (NCT03117738). Although well-tolerated, this approach showed no significant cognitive benefit compared to placebo, with serious adverse events, including pulmonary embolism and esophageal carcinoma. A follow-up study (NCT04482413) is ongoing. In contrast, a parallel trial (NCT05827757) demonstrated that these MSCs can reduce inflammatory cytokines in patients with age-related low-grade inflammation, suggesting an immunomodulatory benefit.

Studies with hUCB-MSCs, such as NEUROSTEM^®^-AD (NCT02054208), showed good tolerability and mild side effects that were resolved quickly. These results, along with related trials (e.g., NCT01297218) [96], support the safety of hUCB-MSCs, with new trials exploring secretomes and extracellular vesicles derived from these cells.

Allogeneic MSC therapies have also shown promise, including Lomecel-B (NCT04040348) and laromestrocel (NCT05233774), both of which demonstrated neurocognitive and neuroimaging improvements. In addition, significant reduction in brain atrophy was noted with laromestrocel. Both therapies are safe, supporting larger-scale trials [97].

Other research includes autologous BMSCs (NCT03724136, NCT02795052) aimed at improving cognitive function as well as amniotic or cord tissue-based therapies (NCT03899298), which are still in early stages. HiPSCs, such as those used in the NCT00874783 study, are being explored for disease modeling and preclinical discovery, offering promising future platforms, although they are not yet suitable for clinical use.

Table 4 summarizes the results and limitations of clinical trials investigating stem cell-based therapies for AD, including autologous adipose-derived MSCs, human umbilical cord blood-derived MSCs, and allogeneic MSCs, highlighting key outcomes such as safety, efficacy, and trial phases.

### 3.3. Emerging Strategies to Enhance Stem Cell Therapies

Despite the challenges of applying stem cells as a treatment against AD, several types of new innovative approaches are being developed to improve their efficacy, specificity, and safety to a significant degree. These emerging strategies hold strong potential to enhance stem cell-based interventions for neurodegenerative diseases, paving the way for more effective and clinically applicable treatments.

The use of genome editing technologies, particularly CRISPR/Cas9, is a viable approach for creating highly precise genetic changes in iPSCs. As discussed in the next chapter, one advantage is the ability to generate isogenic cell lines; this is achieved by introducing or correcting disease-associated mutations without altering the overall genetic background of the individual from whom the cells are derived [98].

CRISPR/Cas9 allows for the creation of more accurate disease models and increases the safety of transplants [99]. Moreover, CRISPR/Cas9 technology can be utilized to knock out oncogenes that are causing tumor growth in iPSCs, thus avoiding teratomas upon implantation, which is a significant concern in cell therapies [100].

The application of stem cell-derived exosomes as a “cell-free” therapy is another form of treatment that does not involve transplanting the stem cells themselves. Exosomes are tiny vesicles secreted by cells that carry molecules that have neuroprotective (neuroprotective functions) and immunomodulatory (modulate the immune system) functions. They can promote the survival and regeneration of nerve cells. The advantage of this strategy is that it does not involve the risks typically associated with whole-cell transplantation, such as immune system rejection by the recipient or uncontrolled cell division. In laboratory experiments using animal models of AD, exosomes obtained from MSCs have been shown to reduce the accumulation of Aβ protein in the brain and improve cognitive function [101].

The use of these scaffold biomaterials is being explored for improving some critical parameters of cell transplantation: transplanted cell survival, their engraftment (i.e., integration into the host tissue), and their proper differentiation into the desired cell types. The scaffolds are three-dimensional materials that attempt to mimic the highly complex structure and composition of the extracellular matrix in the brain. They work by providing structural support and a favorable microenvironment that promotes the incorporation of transplanted stem cells [102]. This increased support enables the therapeutic incorporation and function of transplanted cells in damaged brain disease.

**Figure 3:**
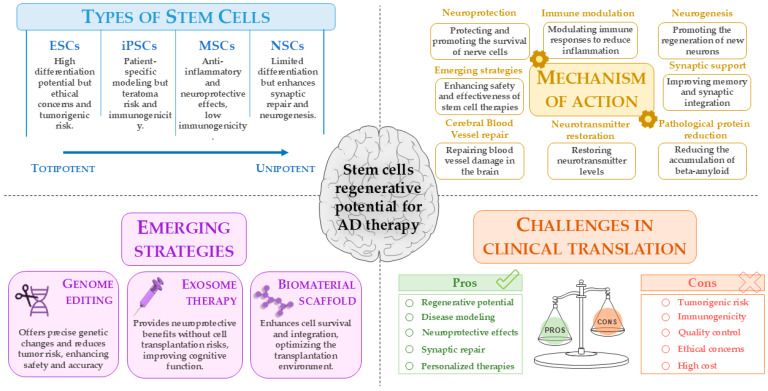
Stem cell-based approaches for AD therapy: opportunities and challenges. This figure summarizes the types of stem cells and their regenerative potential for AD treatment, highlighting mechanisms of action, emerging strategies such as genome editing and exosome therapy, and key clinical translation challenges. While stem cell therapies show promise in neuroprotection, neurogenesis, and synaptic repair, concerns remain regarding tumorigenicity, immunogenicity, and quality control. This image was created using the image bank of Servier Medical Art (Available online: http://smart.servier.com/; accessed on 30 May 2025) licensed under a Creative Commons Attribution 3.0 Unported License (available online: https://creativecommons.org/licenses/by/3.0/, accessed on 30 May 2025). AD: Alzheimer’s disease; ESCs: embryonic stem cells; iPSCs: induced pluripotent stem cells; MSCs: mesenchymal stem cells; NSCs: neural stem cells.

These innovative strategies aim to overcome the current limitations of stem cell therapies for AD using an approach that combines genetic precision, the use of safer cellular components, and optimization of the environment in which the cells are transplanted, potentially leading to more effective treatments for this complex disease (Figure 3).

## 4. Emerging Experimental Models for AD: The Potential of CRISPR/Cas9 and hiPSCs

According to recent studies, hiPSC-derived brain cells represent a valid and reliable in vitro platform for studying cerebral development and functions. These cells can self-organize into functional neuronal networks, displaying electrophysiological properties and pharmacological responses comparable to those observed in mouse models, including an active inhibitory system. hiPSCs represent an emerging approach to AD, mainly used to model the disease and test drugs in vitro. Although not yet clinically applied, studies explore their potential in the preclinical setting, with the future goal of using them in personalized transplantation therapies. However, it must be emphasized that in vitro and/or in vivo models cannot fully replicate the structural and functional complexity of the human brain [103,104].

Currently, available data do not allow us to determine whether iPSCs or hESCs are intrinsically superior for the study of AD. However, iPSCs offer the key advantage of being derived directly from patients with specific genetic mutations, allowing the generation of cellular models that reproduce the genetic background of the disease [105]. Neurons and other brain cells generated from patient-derived cells provide a human-relevant context for studying AD, especially its familial or genetic forms [106]. Unaffected control iPSCs can serve as a basis for introducing mutations via genetic knock-in methods [107,108]. Moreover, iPSCs overcome the ethical issues linked to hESCs, as they are generated from adult somatic cells without involving embryos, facilitating the development of new therapeutic strategies. Recent evidence suggests that using CRISPR/Cas9 to introduce AD-associated mutations in stem cell-derived models enables researchers to study genetic variations more effectively, shedding light on the molecular mechanisms of AD [109]. Findings revealed that the combination of iPSCs and CRISPR/Cas9 is a promising approach for assessing pathological features typical of AD, including protein aggregation, oxidative stress, and neuronal death [110]. However, as we will see in the next chapter, several methodological limitations must be carefully considered. iPSCs, although offering a relevant human model, present challenges related to inter- and intra-lineage variability, epigenetic memory of the tissue of origin, and potential genomic alterations acquired in culture, including tumorigenesis. Two-dimensional models often do not reproduce complex phenotypes such as neurodegeneration or tau pathology, while more mature and physiologically relevant 3D organoids suffer from heterogeneity, lack of vascularization, and limited representation of cells such as microglia. Generation of specific neuronal subtypes or multicellular co-cultures also remains technically challenging and poorly standardized. With respect to genome editing, CRISPR/Cas9 carries inherent risks such as off-target effects, mosaicism, and DNA damage response. Efficient delivery of editing components into post-mitotic neurons remains a challenge, as does the generation of precise knock-in mutations. Furthermore, lengthy protocols, use of antibiotics and plasmids, and analytical biases in single-cell data further complicate the interpretation of results [see Section 5].

This chapter outlines recent advances in AD research combining hiPSCs with CRISPR/Cas9 gene editing.

### 4.1. Building an AD Model Using CRISPR/Cas9 and hiPSCs

CRISPR/Cas9 technology is increasingly used to reduce genetic variability within the same cell types through precise genome editing. In the case of AD research, the use of stem cells facilitates genomic modifications that help uncover specific pathological alterations linked to mutations, allowing for a clearer understanding of how these genetic changes contribute to disease progression.

In brief, iPSCs from patients or healthy individuals are grown under specific conditions to keep them able to change into different types of cells [111,112]. Then, hiPSCs are dissociated with reagents like Accutase, and ROCK inhibitors are added to the medium to optimize conditions [113,114,115,116]. Quality control on iPSC lines is crucial and should be conducted by verifying pluripotency through markers, such as Oct4 and Sox2, and performing karyotype analysis to identify chromosomal abnormalities [107,117].

A sgRNA targeting specific genomic regions is introduced into human iPSCs using nucleofection, transfection, or lipofection methods in conjunction with CRISPR/Cas9 technology [113,118,119,120]. Notably, online tools facilitate the selection of high-specificity sequences, and utilizing Cas9 “nickases” has been shown to reduce off-target effects by cutting one DNA strand, allowing for more precise modifications [119]. Then, sgRNA sequences are carefully inserted into specific plasmid vectors and checked using Sanger sequencing to confirm proper insertion [118].

After CRISPR/Cas9 delivery, clonal expansion of hiPSCs using fluorescence-activated cell sorting or pharmacological selection facilitates the isolation of edited cells, which is important for obtaining isogenic lines [116,121,122]. Thus, edited cells are genetically confirmed using PCR and Sanger sequencing to verify that insertion or deletion has occurred and to determine their status (homozygous, heterozygous, or unmodified) [109]. A controlled and physiologically appropriate environment is then created, in which the properly modified hiPSCs are differentiated into brain derivatives, such as neurons or brain organoids, which can better mimic neurological systems [123,124]. In general, the evaluation of biological and molecular processes of cells under analysis exploits multi-omic techniques that allow a better understanding of the mechanisms related to the disease [125]. Functional validation allows, indeed, a detailed characterization of the genetically modified cells, allowing the comparison between healthy and pathological states and ensuring a more repeatable and ethical model of neurodegenerative disorders [113].

**Figure 4:**
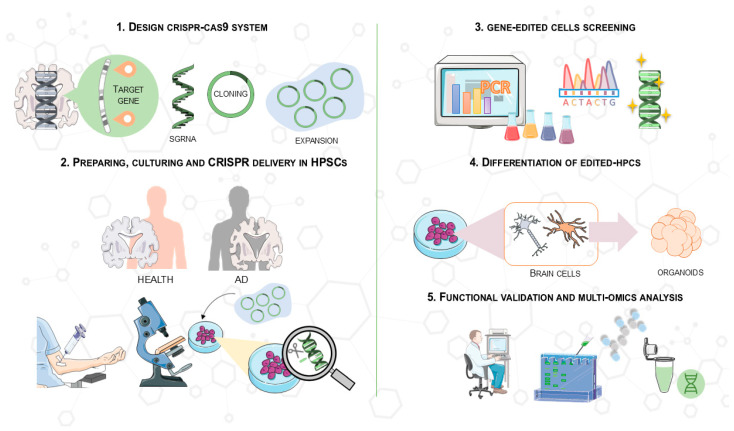
Overview of the CRISPR/Cas9 protocol used in hiPSCs for drug screening and clinical application in AD models. This process involves (1) synthesizing an optimal sgRNA targeting an AD-related gene (e.g., APOE) and cloning it into a CRISPR/Cas9 vector; (2) deriving and culturing hPSCs from patients or controls, followed by CRISPR/Cas9 delivery (e.g., transfection, electroporation); (3) confirming genetic modifications in expanded clones using PCR and Sanger sequencing; (4) differentiating gene-modified hPSCs into brain cells and generating 3D organoids; and (5) investigating molecular pathways and pathological mechanisms to study the effects of genomic modifications in hPSCs. This image was created using the image bank of Servier Medical Art (Available online: http://smart.servier.com/; accessed on 30 May 2025) licensed under a Creative Commons Attribution 3.0 Unported License (available online: https://creativecommons.org/licenses/by/3.0/, accessed on 30 May 2025). CRISPR/Cas9: Clustered regularly interspaced short palindromic repeats/CRISPR-associated protein 9; AD: Alzheimer’s disease; ESCs: embryonic stem cells; hiPSCs: human induced pluripotent stem cells.

A schematic of this process is shown in Figure 4, which illustrates the main steps of CRISPR/Cas9 gene editing in hiPSCs. These steps include the synthesis of specific sgRNAs targeting AD-related genes, the editing of hiPSCs using CRISPR/Cas9 delivery, confirmation of genetic modifications, differentiation into brain cells, and the generation of 3D organoids for functional analysis. This protocol allows for precise modeling of AD in vitro, facilitating drug screening and the study of pathological mechanisms.

### 4.2. The Rise of hiPSCs for Disease Modeling of AD

Gene editing can include different techniques to investigate the pathological mechanisms of AD in iPSC-derived cells [126,127]. The development of human neurons with disease-associated phenotypes helps researchers to uncover critical pathways in AD pathology and model genetic risk factors and protective variants beyond amyloid and tau [25]. As mentioned above, one strategy is the knock-in or the correction of specific mutations in AD-associated genes. A main advantage is the ability to reproduce in vitro models of FAD and associated risk variants, improving the study of pathological characteristics that are difficult to analyze [128]. Recent evidence indicates that cerebral organoids derived from iPSCs provide a more complex and physiologically relevant model for AD. This advancement addresses the limitations of 2D cultures, which fail to replicate intricate cell–cell interactions and do not adequately represent the accumulation of extracellular aggregates [117]. Compared to transgenic mouse models of AD, organoids offer several unique advantages. While mouse models have been invaluable for elucidating disease mechanisms, they often do not fully replicate the human-specific features of AD pathology, such as the exact sequence and structure of Aβ peptides, species-specific tau isoforms, and differences in immune responses. Organoids, being derived from human cells, overcome these species-related limitations and allow the modeling of AD in a human genetic and epigenetic context. Three-dimensional brain organoids have become a powerful tool in modeling AD, offering insights into the molecular mechanisms underlying AD. These organoids replicate the cellular environment of the brain and provide a promising platform for studying disease progression and therapeutic interventions. To enhance their relevance to AD research, it is crucial that 3D brain organoids account for spatiotemporal factors, such as the timing of disease onset (early or late- onset) and the aging process. Early- and late-onset forms of AD present distinct pathological and molecular characteristics, which can be modeled by manipulating the timing of cellular and molecular changes in organoids. Furthermore, since AD is strongly associated with aging, it is essential to incorporate age-related cellular changes into the organoid models [129]. This approach would allow researchers to better understand how the progression of the disease is influenced by age and the timing of onset, ultimately advancing therapeutic strategies tailored to these variations. When combined with CRISPR/Cas9, organoids allow researchers to introduce or correct AD-related mutations and study their effects in a 3D human cell context [130]. Combining this with CRISPR/Cas9 technology and iPSCs to create isogenic cell lines during brain organogenesis offers the ability to generate highly specific models to explore the molecular and cellular mechanisms behind AD [131], with an added advantage of generating a spatiotemporal transcriptomic atlas to map how genetic and epigenetic factors influence brain development and disease progression [132].

Such an atlas would provide insights into how genetic and epigenetic factors contribute to AD progression, including the effects of early- and late-onset AD and aging. By combining iPSCs, CRISPR/Cas9, and spatiotemporal transcriptomics, these models replicate key pathological features of AD, offering a more accurate representation of human disease mechanisms compared to traditional animal models [133,134].

This approach advances the development of more personalized therapeutic strategies for AD. In addition to considering spatiotemporal factors, it is crucial to recognize the role of key signaling pathways, such as Wnt, Shh, BMP/TGF-β/SMAD, and Notch, in shaping the spatial domain identity during brain organogenesis. These signaling pathways are integral to the regulation of cell fate specification, tissue patterning, and the establishment of brain region-specific identities. For example, Wnt signaling plays a critical role in regulating neuronal differentiation and axial patterning, while Shh signaling is pivotal in the patterning of the forebrain and the differentiation of specific neuronal populations. Similarly, BMP/TGF-β/SMAD signaling governs the differentiation of neural progenitors and neuronal specification, whereas Notch signaling regulates neurogenesis and the maintenance of neural stem cells. These pathways interact in complex ways to establish the spatial and temporal organization of the brain. By incorporating these signaling networks into 3D brain organoids derived from iPSCs, researchers can better model how developmental cues influence cellular differentiation and tissue architecture [135].

Manipulating these pathways in organoids can help mimic the spatiotemporal dynamics of the brain in both health and disease, offering a more accurate platform for studying neurodegenerative diseases like Alzheimer’s. Furthermore, by combining these models with CRISPR/Cas9 technology, it is possible to create isogenic cell lines that can be used to explore the specific effects of genetic mutations on the expression and function of these key signaling pathways in a human context.

The potential of these models lies in the ability to generate several human cell populations relevant to AD from a controllable source, the iPSCs, overcoming existing limitations. Thus, the roles of individual brain cell types and their interactions can be studied, including in co-culture models that better mimic neurodegenerative disease [136]. In particular, exploring the role of glial cells can help elucidate pathological features, such as ROS production [136]. For instance, studies using iPSC-derived microglia have shown the crucial regulatory roles of the CX3C chemokine receptor 1 in inflammation and phagocytosis, highlighting its potential for studying neuron–glia interactions during disease [137]. An efficient and reproducible method has recently enabled the generation of iPSC-derived microglia with characteristics closely resembling primary microglia. Haq I. et al. generated a CRISPR ON/OFF system for accurate temporal gene control, without inducing DNA double-strand breaks that can cause genomic rearrangement [120].

Moreover, the ability to recreate specific disease phenotypes in a human context makes these models more predictive of drug efficacy than traditional preclinical models. Key clinical characteristics of AD are replicated in cerebral organoids produced from sporadic individuals, who generally have higher amyloid and tau levels [117]. Notably, organoids with specific mutations, such as PSEN2^N141I^, have shown responses to drugs that increase neuronal activity, indicating their potential to assess the efficacy of new or existing drugs [112].

#### Isogenic Lines in CRISPR/Cas9 and hiPSC-Based Models: Enhancing Precision in AD Research

One of the main challenges in human disease modeling is the genetic variability among individuals, which can complicate the interpretation of experimental results. In the context of neurodegenerative diseases, the creation of iPSCs from patients or healthy individuals has been proposed as a potential strategy to study the effect of a specific genetic modification [138]. Gene editing techniques further enhance this approach by enabling the generation of isogenic lines, genetically identical cells that differ only in the variant of interest, allowing direct comparisons regardless of whether they originate from a patient or a healthy donor [139]. Comparing an edited line to its isogenic control boosts confidence that observed differences are due to specific genetic manipulation, not other genetic variations. For instance, Ng, B. et al. generated isogenic tau-depleted iPSC lines from two healthy individuals using CRISPR/Cas9, which were further differentiated into cortical neurons. Tau-depleted neurons showed decreased activity and neurite outgrowth in comparison to controls. In contrast, Aβ-induced toxicity was reduced, including neurodegeneration, hyperactivity, and deficiencies in mitochondrial transport. These results support chronic tau-lowering strategies as a potential treatment for AD [140], validate the use of animal models such as Mapt^−/−^ mice, and underline tau’s role in Aβ-driven pathology. Similar findings were reported by another group, further supporting tau-lowering strategies in AD [141].

The use of CRISPR/Cas9 to generate genetically engineered hiPSC lines with identical genetic backgrounds represents a significant advance in biomedical research, providing highly controlled and reproducible disease models [112]. The main result is a reduction in genetic background due to variability between different individuals [142]. Another study used iPSC-derived cerebral organoids from two FAD patients carrying the PSEN1^E280A^ mutation to investigate the protective role of the APOE3 Christchurch variant. By introducing or removing APOE3Ch through gene editing, the researchers created isogenic-like comparisons that allowed them to isolate the effects of APOE3Ch on disease pathology in a 3D human brain model [143]. APOE is a major genetic risk factor for late-onset AD, although its pathological role across different brain cell types remains unclear. Notably, CRISPR/Cas9-mediated APOE deletion appears to prevent cellular senescence, highlighting APOE as a promising therapeutic target in aging-related diseases like AD [144]. The introduction of hiPSCs combined with CRISPR/Cas9 technology has enabled the generation of isogenic lines carrying either the APOE3 or APOE4 allele, thereby allowing for a precise attribution of observed phenotypes to the specific variant under study [125]. Differentiation of these lines into neurons, astrocytes, and microglia revealed that APOE4 promotes early neuronal maturation, increases Aβ42 secretion, alters astrocyte lipid metabolism, and impairs glial amyloid clearance. In brain organoids, APOE4 leads to the accumulation of Aβ and phosphorylated tau, although with slower kinetics compared to FAD models [118]. Importantly, genomic conversion of APOE4 to APOE3 attenuated most of these phenotypes, confirming the central role of APOE4 in sAD pathology [145].

Another critical example involves different modifications of the *APP* gene, which is pivotal in AD pathology. Specifically, iPSC cells harboring the *V717I* (London) and *KM670/671NL* (Swedish) mutations into the *APP* gene, commonly used in animal models such as 5xFAD, showed increased Aβ production similar to AD patients [146]. Researchers applied CRISPR/Cas9 to edit the *CRTC1* gene in hiPSC with the APPSwe mutation, enhancing neuronal functionality. The results were also reproduced in vivo, as injection of the modified *CRTC1* into 5xFAD mice restored synaptic plasticity, crucial for memory [147]. Notably, gene knockout models of APP highlighted defects in neuronal development and synaptic function, implicating cholesterol transport and distribution in neurons [148]. Furthermore, Ye, T. et al. demonstrated that correcting *APP* gene dosage using paired Cas9 nickases in hiPSC-derived neurons reduced Aβ production, Tau hyperphosphorylation, and neuronal loss [119].

Researchers used hiPSC and CRISPR/Cas9 technology to study whether the loss of the *SORL1* gene, which is involved in APP cellular trafficking, causes problems in early endosomes in neurons and other affected cell types [149]. The pathophysiology of AD is significantly influenced by endosomal trafficking disturbance. Another study revealed that rare mutations in the *SORL1* gene could disrupt the maturation and trafficking of a protein called SorLA in neurons derived from hiPSCs. Due to its retention in the endoplasmic reticulum, these alterations decrease the quantity of SorLA in the endosomal system and plasma membrane, which eventually results in greater release of Aβ [150].

Furthermore, generating a homozygous *ABCA7*-knockout iPSC line using CRISPR/Cas9 gene editing is an effective approach to explore the role ABCA7 loss plays in AD [115]. This iPSC line displays pluripotency markers, maintains a stable karyotype, and can develop into all three germ layers [151]. Moreover, iPSC-derived cortical organoids with *ABCA7* knockout demonstrate that loss of this AD risk gene leads to mitochondrial oxidative stress and apoptosis. *ABCA7* deficiency increased ROS production and activated caspase 3, a key mediator of oxidative stress-induced apoptosis. Upregulation of pro-apoptotic genes such as *APAF1*, *BAK1*, and *XIAP* was also observed, linking ABCA7 loss to impaired mitochondrial lipid metabolism, neuronal apoptosis, and disrupted synapse formation [152].

Overall, there is a growing interest in analyzing the impact of CRISPR/Cas9 and hiPSC technologies. Interestingly, using different gene targets appears useful in generating more accurate models (as summarized in Table 5), allowing the exploration of complex pathogenic mechanisms and potential therapeutic targets. Nevertheless, in the case of AD, these models have significant limitations, as we will see in the next chapter. Creating an in vitro system closely representing the human brain is difficult. In addition to the in vitro models, high spatiotemporal resolution imaging techniques, such as functional magnetic resonance imaging (fMRI), can provide valuable insights into the spatial and temporal dynamics of brain activity in AD. fMRI allows for the real-time monitoring of brain regions involved in cognitive functions and their alterations throughout the disease. By combining fMRI with cellular and molecular models, researchers can gain a more comprehensive understanding of functional changes associated with AD pathology, particularly the progressive nature of amyloid accumulation, tau aggregation, and their effects on brain network connectivity. This approach can aid in better characterizing disease stages, improving diagnostic accuracy, and evaluating the efficacy of therapeutic interventions [153]. Continued progress in this field could play a crucial role in the development of personalized treatments and in improving therapeutic intervention strategies for AD. All these issues highlight the urgent need for more rigorous protocols, validated systems at multiple levels (cell, organoid, animal), and increased standardization to improve the reliability of in vitro models. A more detailed overview of these limitations is provided in Chapter 5 and summarized in Table 5.

## 5. Challenges and Future Directions in CRISPR and hiPSCs-Based Approaches for AD

Stem cell-derived models, especially those based on hiPSCs and hESCs, have significantly advanced AD research by enabling the study of human neurons in vitro. These models facilitate the investigation of genetic mutations and disease mechanisms in a controlled environment, overcoming some limitations of animal models. Despite their great potential, hiPSC-derived models, often combined with CRISPR/Cas9-based genetic engineering to study AD-associated mutations, still face significant limitations that define areas for future development [103,118,152]. However, there is not yet a shared reference line, as in the case of C57Bl/6J mice, mainly because a thorough and comparative analysis of the different lines has not yet been conducted [154].

Moreover, the high cost of specialized reagents, consumables, sophisticated equipment, laborious protocols, extensive quality controls, and model complexity significantly increase the costs of research using isogenic hiPSC-based and CRISPR/Cas9 lines. Furthermore, in addition to regulatory complexity, ethical considerations regarding the application of hiPSCs and CRISPR/Cas9 technology in AD research are currently under discussion. The potential for germline leakage, unintentional edits in reproductive cells, raises long-term safety questions that cannot yet be fully addressed with current technologies [155]. Furthermore, informed consent becomes more nuanced in the context of irreversible genome editing or experimental stem cell transplants. Thus, while CRISPR- and iPSC-based models offer transformative potential, their clinical adoption requires not only technical refinement but also robust frameworks for safety monitoring, ethical oversight, and fair distribution.

While in the previous chapter we explored the potential in more detail, here we will evaluate the major limitations currently present in the CRISPR/Cas9 and hiPSC-based disease modeling.

### 5.1. Bioethical Implications of Stem Cell and Genome Editing Technologies in AD

Despite encouraging preclinical results, the real-world clinical translatability of CRISPR/Cas9 and stem cell-based approaches for AD remains constrained by several regulatory, technical, and ethical barriers. A critical distinction must be made between laboratory efficacy, often demonstrated under highly controlled in vitro conditions, and the rigorous requirements for clinical approval. Regulatory bodies such as the U.S. Food and Drug Administration (FDA) and the European Medicines Agency (EMA) require comprehensive data on safety, reproducibility, and long-term outcomes before authorizing trials in humans [156]. Detailed characterization of the product, including vectors and modified cells, is mandatory, and high standards of quality, purity and traceability must be guaranteed. The FDA’s guidance on somatic genome editing emphasizes the need for validated delivery methods, detailed off-target analysis, and long-term follow-up, particularly in sensitive tissues like the brain. Similarly, the EMA classifies CRISPR-based and stem cell therapies as Advanced Therapy Medicinal Products, requiring extensive quality controls, traceability, and compliance with Good Manufacturing Practices [157,158].

First, the origin of human stem cells remains a foundational ethical issue. While the use of iPSCs, reprogrammed from somatic cells, circumvents some of the more controversial ethical debates associated with ESCs, the derivation, storage, and use of human biological material still require strict ethical oversight and adherence to national and institutional regulations. The use of patient-derived hiPSCs for disease modeling further emphasizes the need for responsible handling of human tissue and transparency in research protocols [105].

Second, the application of CRISPR/Cas9 for genome editing in human cells raises broader ethical questions regarding the manipulation of the human genome. In the reviewed literature, genome editing is primarily employed to generate disease models by introducing or correcting specific mutations in hiPSC lines. While these modifications are confined to in vitro research and potential somatic (non-heritable) therapies, they nonetheless prompt ongoing debate over the moral boundaries of altering human genetic material. Concerns grow sharper when considering future therapeutic applications, where safety, long-term consequences and broader societal impacts must be considered [103,114,142,143].

A key concern is technical safety, particularly the risk of off-target effects, which are unintended genetic alterations outside the intended site. This poses ethical dilemmas, especially in therapy, as such modifications could lead to harmful outcomes. Using isogenic controls helps minimize variability and identify off-target effects, but bioethical issues surrounding CRISPR/Cas9 remain unresolved. Comprehensive global regulations, developed with input from various stakeholders, are essential for safe and responsible use [159].

Lastly, the issues of informed consent and data privacy are central when working with iPSCs derived from patients with identifiable conditions. The guidelines for reporting research require specifying the measures taken to ensure reproducibility, randomization and blinding of experiments, which are fundamental aspects of research ethics [144]. For example, studies involving rare genetic variants such as the APOE3 Christchurch mutation inherently involve sensitive genetic information. While the primary literature may not always detail the consent procedures, ethical research practice demands comprehensive informed consent processes and careful attention to participant privacy, especially when genomic data are involved [143]. Another critical issue is equitable access: these therapies involve high development and administration costs and may not be readily available to patients outside of highly specialized clinical centers [160].

### 5.2. Current Limitations of Gene Editing and Stem Cell Technologies in AD Modeling and Therapy Development

#### 5.2.1. Limitations of hiPSC-Derived In Vitro Models for AD

Protocols for differentiating hiPSCs into AD-relevant cell types, such as cortical neurons or microglia, can be time-consuming. Many existing protocols for differentiating iPSCs into microglia typically take more than 30 days, some take up to 74 or 75 days. Although shorter protocols exist, some utilize the integration of plasmids and antibiotics that can activate microglia [120]. Additionally, the need to generate specific neuronal subtypes, such as induced glutamatergic neurons, or to obtain specific cell populations (e.g., deep or higher cortical neurons), adds complexity and potentially time to the differentiation process [110].

One major limitation is the reduced biological complexity of in vitro models compared to the human brain. Key cellular components such as mature glial cells, particularly microglia, crucial players in neuroinflammation, are often absent or poorly represented. Even with the incorporation of iPSC-derived microglia, the full replication of in vivo behavior has yet to be demonstrated. Similarly, the lack of vascularization is a critical gap, as vascular dysfunctions are implicated in AD pathology [114,142]. Many models also focus on specific neuronal subtypes, such as glutamatergic neurons, failing to capture the full cellular diversity of the brain [110]. A further challenge is the difficulty of modeling the late-onset and progressive nature of AD, as most in vitro systems cannot reproduce the decades-long disease course or the aging-related phenotypes that characterize the clinical manifestation of the disorder [107]. The relatively short lifespan of in vitro cultures, combined with the developmental immaturity of iPSC-derived cells, limits their utility in modeling long-term disease progression and age-dependent pathological features [138].

Compared to animal models, 2D cell cultures, such as hiPSC-derived neurons, possess a human genetic background, but at the same time have intrinsic limitations and fail to show extracellular deposition of amyloid aggregates [112]. In addition to biological limitations, there is high variability in differentiation outcomes and the composition and maturation of brain organoids [130]. The variability can affect the reproducibility of studies and the efficiency of differentiation, thus standardization of protocols is needed to produce reproducible cell populations at scale [143]. The generation of isogenic iPSC lines involves several steps, including designing and introducing CRISPR components, selecting edited cells, subcloning single cells, and expanding clones. It is essential to inspect the clones for the desired edits and verify that they maintain a normal karyotype and express pluripotency markers [117]. The results obtained from in vitro models based on CRISPR/Cas9-edited iPSCs often require external validation in more complex systems, such as co-cultures including different cell populations, such as mature glia, or in vivo animal models, to confirm their physiological relevance and therapeutic potential [148]. Therefore, producing iPSC-based models, especially complex brain organoids, at a scale sufficient for high-throughput drug screening or future clinical applications presents scalability challenges. Another important limitation of hiPSC-based models is that they do not fully capture the aging process, which plays a central role in the development and progression of AD, even in cases caused by genetic mutations like those in the APP gene. Because iPSCs are reprogrammed into a youthful, embryonic-like state, they lose many of the typical features of aged cells, such as DNA damage, mitochondrial dysfunction, and impaired protein regulation. These age-related changes are crucial to understanding how the disease unfolds over time [161,162]. For this reason, long-lived systems like animal models are still essential for studying the mechanisms that emerge with aging and for testing treatments that need to work in the context of an aging brain.

#### 5.2.2. Limitations and Challenges of CRISPR/Cas9 Technologies in AD Research

While CRISPR/Cas9 is a powerful tool for precise genetic modification, the challenges of ensuring that there are no unwanted changes and achieving efficient and safe delivery to brain tissue remain crucial obstacles to its full clinical application in AD.

Currently, there are no active, publicly registered human clinical trials using CRISPR/Cas9 technology to treat AD. The most advanced research is still in the preclinical or animal model development phase, with promising results but not yet in human clinical trials.

Clinical trials must be designed to monitor pharmacodynamics, pharmacokinetics, and duration of effects, with long-term follow-up to detect any late adverse effects. Furthermore, accurate informed consent is essential, given the innovative nature and ethical risks (such as possible hereditary alterations), and policies that promote equitable access to therapies, which are often expensive and technologically complex. These conditions make the clinical application of CRISPR/Cas9 in neurodegenerative diseases such as AD particularly challenging [163].

A significant concern for the therapeutic use of CRISPR/Cas9 is the unwanted genetic modifications that may occur at sites other than the intended target, with unexpected consequences [107]. Cas9 nucleases can induce a substantial number of off-target mutations in human cells, underscoring the need for rigorous validation and safety evaluation [112]. Although the use of isogenic controls helps to minimize potential off-target effects, the possibility of their existence cannot be completely excluded [143]. Efforts to improve specificity using high-fidelity Cas9 variants, base editing, and prime editing are ongoing [109,150]. For instance, using the coupled Cas9 nickase significantly reduces off-target effects, although it may have a lesser impact on gene editing efficiency and targetability [119]. Furthermore, generating knockout cells or introducing specific mutations often results in relatively low success rates, making these approaches impractical for many laboratories. Alteration of essential genes, critical for maintaining pluripotency and promoting differentiation of specific cell lineages, can significantly impact the outcomes of functional studies. Inducible Cas9 systems, such as those regulated by doxycycline, represent a potential solution to this challenge, allowing temporal control of Cas9 expression and genome editing [107,109].

The manipulation of large-scale gene expression, which is essential for understanding the molecular role of AD-related genes, is challenged by the low insertion efficiency of CRISPR/Cas9 vectors and high cell mortality. As a result, the use of CRISPR/Cas-mediated gene editing in cell pool experiments is limited [25]. Moreover, although not specifically related to the CRISPR/Cas9 mechanism itself, the ethical and legal implications of gene editing pose significant challenges, particularly for clinical applications and translation of the technology. This requires thorough validation before clinical trials can proceed.

### 5.3. Future Direction

Despite the transformative potential of CRISPR/Cas9 and hPSCs for AD research and therapy, challenges related to safety (e.g., off-target) and standardization, coupled with the complex ethical and legal considerations of human gene editing and stem cell use, represent significant difficulties that must be addressed before these technologies can be widely translated into effective clinical interventions for AD [105,143]. Moreover, the clinical application of hiPSC-derived technologies, often integrated with gene editing such as CRISPR/Cas9, and the associated ethical considerations, present several significant challenges. As mentioned in Chapter 3, the survival and functional integration of transplanted cells in the often hostile environment of the brain affected by neurodegenerative diseases is a fundamental challenge for therapies based on cell transplantation.

Future research efforts are expected to focus on overcoming the current limitations of CRISPR/Cas9 and stem cell-based models for AD. One of the key priorities is the development of more complex and physiologically relevant systems, including brain organoids and co-culture models that incorporate mature microglia, other glial cell types, and vascular structures [112,117,118,136]. Although significant progress has been made with iPSC-based models in bypassing the differences between different organisms, limitations remain, particularly regarding the potential loss of age-dependent cellular phenotypes. Therefore, integrating in vitro results with data from in vivo studies and human brain tissue will further strengthen the translational relevance of these models [107,148]. A valid choice could be the use of the KOLF2.1J hiPSC subline, which has obtained good results. Several experiments have shown that KOLF2.1J cells can maintain genetic stability; even after multiple CRISPR/Cas9 modifications, these cells are free of variants that could compromise neuronal function [154].

Expanding research to include a broader range of neuronal subtypes and investigating cellular interactions using multi-omic approaches may also uncover novel mechanisms and therapeutic targets [125]. For instance, edited iPSC-derived models can be used to investigate the impact of genetic variations on specific cellular processes, such as oxidative stress, mitochondrial function, the endolysosomal system, proteostasis, synaptic plasticity, and signaling pathways (e.g., Wnt/β-catenin) [114,143,152]. These advancements aim to better recapitulate the cellular diversity and interactions present in the human brain. However, improving the modeling of aging and disease progression remains a critical goal, with strategies being explored to induce age-related phenotypes or extend culture duration to capture long-term neurodegenerative processes. The development of more disease-relevant functional assays capable of capturing specific pathological features of AD and evaluating long-term treatment effects represents an important step forward [143]. Three-dimensional cultures are becoming increasingly recognized as more relevant to disease models compared to 2D cultures, as they provide an environment that more closely resembles the human brain, allowing for better reproduction of complex cell-cell interactions and disease phenotypes [114]. Future research efforts should continue to employ these models to evaluate the efficacy of novel drug candidates or to explore the repositioning of existing approved therapies [112]. Enhancing the robustness and reproducibility of differentiation protocols, alongside the standardization of functional assays, will be essential to reduce variability between cell lines and experimental batches, thus improving the reliability of findings across studies [103]. Furthermore, the combination of organoid-based models with computational analysis and mathematical modeling offers a strategic approach for advancing precision medicine in the treatment of AD [117]. Overall, the ability to evaluate neuronal activity and its responses to drugs makes organoids valuable tools for drug screening efforts [112].

Moreover, future work should continue to refine functional validation strategies following genetic editing, ensuring that the biological impact of introduced mutations is thoroughly characterized. Although isogenic lines reduce the potential off-target effects of CRISPR technology, future studies are needed to further investigate their utility for functional validation and therapeutic screening. Furthermore, manipulation of gene dosage, such as creating heterozygous knockout cells that better mimic partial reduction of gene expression, is not currently rapid and efficient in iPSCs, indicating an area for future methodological development [143].

**Figure 5:**
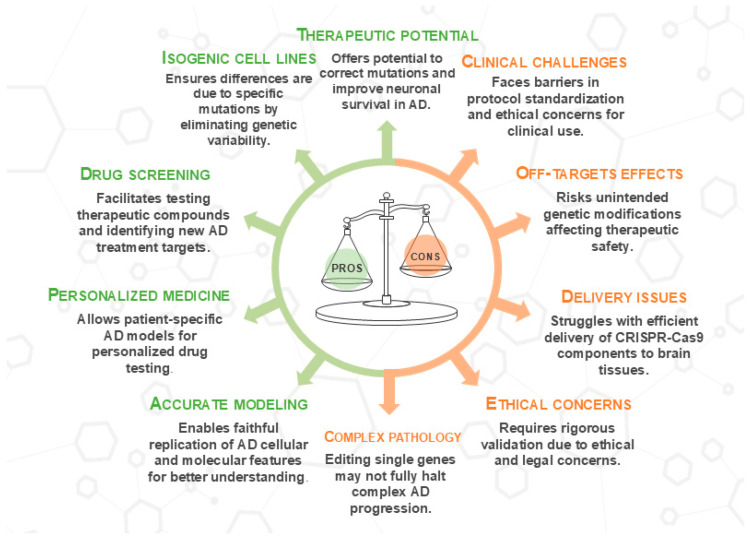
Advantages and disadvantages of combining CRISPR/Cas9 and stem cells for AD. The figure schematizes the key points of using CRISPR/Cas9 technology and stem cells in AD research. Advantages (PROS) are shown on the left, indicated by green arrows, while disadvantages (CONS) are on the right, marked by orange arrows. This image was created using the image bank of Servier Medical Art (Available online: http://smart.servier.com/; accessed on 30 May 2025) licensed under a Creative Commons Attribution 3.0 Unported License (available online: https://creativecommons.org/licenses/by/3.0/, accessed on 30 May 2025). CRISPR/Cas9: clustered regularly interspaced short palindromic repeats/CRISPR-associated protein 9; AD: Alzheimer’s disease.

As illustrated in Figure 5, the balance between the advantages and limitations of CRISPR/Cas9 and iPSC-based models in AD research remains delicate. Despite these continued constraints, the integration of CRISPR/Cas9 with hiPSC-based systems remains of great promise. The ever-growing body of in vitro and in vivo evidence underlines the viewpoint that, ultimately, the therapeutic benefits may transcend the current technological and ethical constraints. The figure reflects the optimistic perspective that the potential benefits may outweigh the current limitations, especially in the long term.

## 6. Conclusions

The combination of CRISPR gene editing technology, stem cell-based models, and neuroengineering technologies is revolutionizing AD research and therapeutic development. CRISPR/Cas9 enables precise modeling of pathogenic mutations in human-relevant systems, such as patient-derived neurons and cerebral organoids. These platforms are essential for studying key AD mechanisms, including Aβ and tau aggregation, mitochondrial dysfunction, synaptic alterations, and neuroinflammation. The use of isogenic cell lines minimizes background variability, allowing direct attribution of phenotypic differences to specific genetic edits. The combined approach allows for creating specific genetic models by introducing or correcting AD-associated mutations in genes such as *APP*, *PSEN1*, *PSEN2*, *APOE*, *SORL1*, *ABCA7*, and others, providing insights into the mechanisms underlying disease progression. However, the mechanisms driving tau pathogenesis may involve additional factors, including epigenetic modifications that influence tau expression and phosphorylation. These modifications may represent a potential mechanistic gap, with epigenetic regulation playing a role in how tau is modified independently of the genetic mutations driving Aβ accumulation. Further studies into epigenetic regulation may provide insights into this discrepancy.

While numerous CRISPR/Cas9-based studies and hiPSC-derived models have significantly advanced our understanding of AD pathology, critical evaluation of their limitations is essential for interpreting their translational value. Although genetic targets such as *APP*, *PSEN1*, *MAPT*, and *APOE* have shown promise in modulating core disease pathways, most findings remain confined to in vitro systems or preclinical models, with limited validation across multiple platforms. Similarly, while iPSC-derived neurons, astrocytes, and microglia enable patient-specific modeling and functional exploration, they present variability, incomplete maturation, and challenges in replicating aging-related phenotypes. While 3D brain organoids are more faithful models of human AD pathology compared to 2D cultures, they still suffer from batch-to-batch heterogeneity and incomplete cellular complexity, which must be considered when extrapolating findings for therapeutic development. Despite promising preclinical results, the clinical translation of these findings remains complex, and their true potential will depend on the continued refinement of modeling systems and integration of cross-validation strategies to bridge the gap between experimental insight and clinical application. No active clinical trials currently apply CRISPR/Cas9 directly to AD, largely due to the disease’s multifactorial nature, challenges in CNS-targeted delivery, and unresolved safety concerns, particularly regarding off-target effects and long-term outcomes. Rigorous standards for vector quality, genomic integrity, and follow-up underscore the need to bridge the gap between laboratory feasibility and therapeutic approval are required. Similarly, stem cell therapies have demonstrated safety in early-phase trials, but with limited cognitive benefit to date. More refined cell sources (e.g., hiPSC-derived neural progenitors) and delivery strategies may offer enhanced efficacy but will need careful validation in controlled settings. Ultimately, while gene and stem cell-based therapies hold significant potential for AD, a cautious and critically informed approach is essential. Future research should prioritize translational rigor, standardized outcome metrics, and long-term safety monitoring to responsibly bridge the gap from innovation to clinical application.

## Figures and Tables

**Table 2:** Gene therapy approaches for AD and related dementias. Summary of major clinical trials of gene therapy in AD, amyloid transthyretin amyloidosis, and FTD-GRN, including CRISPR/Cas9 and AAV vectors for NGF, BDNF, human telomerase reverse transcriptase, and progranulin. Preliminary results show good safety and therapeutic potential, despite limitations in targeting and tolerability.

Therapy/ Vector	Target/Gene	Disease	Phase	Outcomes/Mechanisms	Limitations/AEs	NCT
NTLA-2001	Transthyretin	Hereditary amyloid transthyretin amyloidosis	Phase 1	In vivo CRISPR/Cas9 editing reduced transthyretin protein levels significantly and durably	Mild, transient adverse events; proof-of-concept for gene editing in humans	NCT04601051
CERE-110 (AAV2-NGF)	NGF	AD	Phase 1/2	Sustained gene expression; axonal sprouting and hypertrophy	Limited vector uptake and targeting; modest clinical effect	NCT00087789, NCT00876863
AV-hTERT	Human Telomerase Reverse Transcriptase	AD	Phase 1	Potential lifespan extension of neurons; neuroprotection hypothesis	Pending results	NCT04133454
AAV2-BDNF	BDNF	AD/Mild Cognitive Impairment	Phase 1	Neurotrophic support; goal to slow neurodegeneration	Pending results	NCT05040217
R006	Progranulin	FTD-GRN	Phase 1/2	Increased progranulin in cerebrospinal fluid; improved lysosomal function in models	Cerebrospinal fluid pleocytosis; transient neurofilament light chain; dorsal root ganglia toxicity	NCT04408625
AVB-101	Progranulin	FTD-GRN	Phase 1	Aims to restore progranulin; mechanism like PR006	Results pending	NCT06064890

AD: Alzheimer’s disease; FTD-GRN: frontotemporal dementia with granulin mutation; NGF: nerve growth factor; AAV: adeno-associated virus; BDNF: brain-derived neurotrophic factor; CRISPR: clustered regularly interspaced short palindromic repeats; Cas9: CRISPR-associated protein 9.

**Table 3:** Characteristics and therapeutic potential of stem cells in neurological disorders.

Stem Cell Type	Origin	Potential for AD	Mechanisms of Action	Advantages	Limitations	Ref.
Totipotent Stem Cells	Zygote (1–8 cells)	Theoretical, but not used in practice	Can generate all cell types, including extraembryonic tissues	Maximum plasticity	Ethical concerns; not used in research due to developmental role	[68,69]
Pluripotent Stem Cells (ESCs, iPSCs)	Blastocyst (ESCs), reprogrammed somatic cells (iPSCs)	High potential; ESCs and iPSCs can differentiate into neural cells	Differentiate into neurons, replace lost cells, provide neurotrophic support, reduce inflammation	High versatility; genetic modeling with CRISPR/Cas9	Risk of uncontrolled growth (tumor formation), ethical issues (ESCs), immune rejection	[70]
Multipotent Stem Cells (MSCs, NSCs)	Adult tissues (e.g., bone marrow, CNS)	Moderate potential; MSCs and NSCs have been studied in AD models	Modulate immune response, promote neuronal survival, release neurotrophic factors	Immunomodulation, trophic effect, ease of isolation (MSCs)	Limited differentiation capacity, variability in clinical outcomes, potential immune response	[77,84]
Oligopotent Stem Cells	Hematopoietic or glial tissues	Limited potential; can generate specific cell types, but not widely studied for AD	Generate limited cell types; potential for supporting specific neural functions	Defined specialization	Restricted differentiation potential, limited applications in neurodegenerative diseases	[72]
Unipotent Cells	Adult tissues	Single cell type	Muscle stem cells, epidermal stem cells	High safety	Limited application to the central nervous system	[72]

AD: Alzheimer’s disease; ESCs: embryonic stem cells; iPSCs: induced pluripotent stem cells; MSCs: mesenchymal stem cells; NSCs: neural stem cells.

**Table 4:** Clinical trials of stem cell-based therapies for AD: outcomes, limitations, and study phases.

Mechanism/Target	Study Phase	Main Outcomes	Main Limitations	NCT
**Autologous Adipose-Derived MSC**				
Safety and efficacy assessment to improve cognition or slow progression of AD; in the laboratory, it has shown reduction in inflammation	Phase 1, Phase 2	Well tolerated, but serious adverse events (e.g., pulmonary embolism, esophageal carcinoma) and no significant benefit on cognition, behavior, or biomarkers compared to placebo	Very small sample size (7 AstroStem, 5 placebo), with no healthy control group or long-term follow-up; in some cases, faster than expected cognitive decline; no significant cognitive benefit over placebo	NCT03117738
To compare the efficacy and safety of AstroStem versus placebo in patients with mild Alzheimer’s disease	Phase 2	NA	NA	NCT04482413
Prevent age-related inflammation (“inflamm-aging”), associated with diseases such as AD, through the reduction of pro-inflammatory cytokines	Phase 1	Safe and well tolerated; significant reduction of inflammatory cytokines and improvement of immune ratios (IL-4/IL-10, IL-2/IL-10)	Phase 1, single-group, open-label study; primary focus is age-related inflammation, although related to AD	NCT05827757
**Human Umbilical Cord Blood-Derived MSC**				
Evaluate safety, dose-limiting toxicity and exploratory efficacy; preclinical evidence indicates functional improvements and amyloid reduction	Phase 1, Phase 2	Feasible, safe and well tolerated treatment. Common adverse events were mild and transient; three serious related events, no dose-limiting toxicities. No serious events at 36 months. Efficacy not reported.	The efficacy has been evaluated in an exploratory manner and further studies are needed for a thorough understanding.	NCT02054208
Long-term safety and efficacy follow-up	Phase 1	NA	NA	NCT01297218
**Allogeneic MSCs**				
To investigate the safety, tolerability and efficacy of stem cell therapy for various acute and chronic conditions	Phase 1, Phase 2	NA	NA	NCT04040348
Slowing the clinical progression of AD, brain atrophy, and neuroinflammation	Phase 2	Laromestrocel has shown good safety and clinical improvements, with slowing of brain atrophy and reduction of neuroinflammation	Larger-scale clinical trials of laromestrocel in AD are warranted	NCT05233774
**Bone Marrow MSCs**				
Improve cognitive impairment in AD and other dementias; also evaluate use in combination with near-infrared light	NA (unspecified, recruiting by invitation)	NA	NA	NCT03724136
**Other preliminary studies**				
To determine the safety and efficacy of amniotic tissue and umbilical cord for the treatment of various conditions, including neurological conditions	Phase 1	Not yet recruiting, results not available; the hypothesis is that the treatments are extremely safe and statistically beneficial	NA	NCT03899298
hiPSCs	Recruiting	Develop hiPSCs for disease modeling, drug discovery, and basic research; develop technology that could eventually enable the use of hiPSCs for future transplant therapies	hiPSCs developed in this research are not intended for use in transplant therapy	NCT00874783

MSC: Mesenchymal stem cells; AD: Alzheimer’s disease; CRISPR/Cas9: clustered regularly interspaced short palindromic repeats/CRISPR-associated protein 9; hiPSCs: human-induced pluripotent stem cells; IL: interleukin; NA: not available; NCT: National Clinical Trial.

**Table 5:** Summary of selected studies using CRISPR/Cas9-edited iPSC models to investigate AD. The table includes the aim of each study, the genome editing strategy employed, the specific genetic targets, validation methods, key findings, and reported limitations. References are provided for further details.

Study Aim	CRISPR/Cas9 Editing Method	Genetic Target	Validation	Key Results	Limitations	Ref
Application of CRISPR/Cas9 genome editing in hiPSC-derived neurons to study neurodegenerative disease mechanisms	Genome editing using Cas9–gRNA ribonucleoprotein complexes	Neurodegeneration-related genes (e.g., genes involved in mitochondrial dysfunction, oxidative stress, and synaptic activity)	qPCR, immunocytochemistry, Western blotting, live-cell imaging, functional assays	CRISPR/Cas9 successfully models disease-related mutations, enabling analysis of mitochondrial dysfunction and oxidative stress in patient-derived hiPSC neurons	High variability in differentiation outcomes and challenges in recapitulating late-onset disease phenotypes	[106]
Investigate the impact of the *PSEN1^F105C^* mutation on tau accumulation and mTOR signaling in human neurons using CRISPR/Cas9 gene editing	CRISPR/Cas9 combined with piggyBac transposon was used to introduce heterozygous and homozygous *PSEN1^F105C^* mutations in hiPSCs	*PSEN1^F105C^* mutation, linked to FAD, affecting Akt/mTORC1 signaling and tau pathology	Genomic sequencing, IF, Western blot, ELISA, and functional assays, including autophagy and lysosomal activity measurements	*PSEN1^F105C^* mutation led to increased Aβ production, hyperphosphorylated tau accumulation, and impaired starvation-induced mTORC1 suppression and autophagy; treatment with the mTOR inhibitor Torin1 reduced tau pathology and restored autophagic function	Further studies are needed to validate findings in co-culture and in vivo models to better assess the impact of the mutation on neurodegeneration	[107]
Generate isogenic iPSC lines and provide a versatile tool for studying gene functions, modeling diseases in vitro, and screening drugs in hiPSCs or differentiated cells.	Dox-inducible Cas9 integrated at AAVS1 via TALENs; gRNAs delivered by lentivirus (gRNA + EGFP); editing induced by doxycycline	AAVS1 locus targeted for dox-inducible Cas9 insertion; specific genes edited via gRNAs; *RPS24* gene	Cas9 cassette integration at AAVS1 confirmed with junction PCR (1068 bp product); dox-inducible Cas9 expression verified by Western blot and IF; gene editing validated using T7EI assays	Gene editing efficiency reached ~60% (transfection) and up to 90% (lentivirus); editing occurred rapidly (≥50% in 2–3 days) and was sustained across dox doses; Cas9 expression confirmed with Western blot and IF	Minor limitations include rare random integrations, occasional multicellular wells post-sorting, low single cell survival and clone differentiation after 10 days, mitigated by subcloning and rigorous screening	[109]
Identification of new pathways contributing to iron accumulation in neuronal cells using CRISPR in iPSC-derived neurons	CRISPRi for gene knockdown	Genes involved in iron homeostasis, mitochondrial electron transport chain, autophagy, and GPI synthesis	Bioinformatics analysis, FeRhoNox-1 fluorescence for iron accumulation, KEGG pathway analysis, scRNA sequencing	Identified new molecular pathways linked to iron accumulation, including mitochondrial dysfunction, autophagy impairment, and GPI synthesis defects	Findings are limited to glutamatergic neurons; selective vulnerability of other neuronal types remains unaddressed	[110]
To study the effect of MT5-MMP deficiency in human hiPSCs under physiological conditions using CRISPR/Cas9 to assess neural function and neuron-glia interactions	*MMP24* knockout hiPSCs were generated using CRISPR/Cas9 with a modified PX459 plasmid	*MMP24* gene, encoding MT5-MMP; insertions and deletions within exon 2	MT5-MMP knockout was confirmed with PCR screening and Sanger sequencing of exon 2 indels; loss of *MMP24* expression was validated using qPCR showing minimal mRNA levels in knockout clones	MT5-MMP-deficient neurons showed more and longer neurites, with increased dendritic branching but unchanged mean neurite length and electrophysiological properties; astrocytes showed altered morphology, reduced GLAST expression, and S100β changes in arborized cells; GFAP and ALDH1L1 levels were unaffected	Neuronal immaturity may limit phenotype detection; knockout astrocytes may reduce neuronal support (GLAST); possible developmental delay in astrocytes; data collection not blinded	[111]
Generate and characterize a 3D organoid model of AD using patient-derived hiPSCs with a *PSEN2* mutation and isogenic controls	CRISPR/Cas9 was used to correct the *PSEN2^N141I^* mutation in patient-derived hiPSCs, creating isogenic control lines	*PSEN2^N141I^* mutation, linked to FAD, affects γ-secretase activity and Aβ production	Morphological and molecular characterization, immunostaining, calcium imaging, and functional neuronal activity assays	AD organoids exhibited increased Aβ42/Aβ40 ratio, asynchronous calcium transients, and enhanced neuronal hyperactivity compared to isogenic controls, mimicking AD pathology	The model lacks microglia and vasculature, limiting full AD pathology replication. Further validation in co-culture systems or in vivo models is needed	[112]
Investigate the role of DPYSL2-B in neurodevelopmental disorders by creating a CRISPR/Cas9 knockout model in hiPSC-derived glutamatergic neurons	CRISPR/Cas9 editing of DPYSL2-B’s first exon via sgRNA and lipofection	DPYSL2-B, linked to mTOR signaling and schizophrenia	Sanger sequencing confirmed successful knockout; RNA-seq and Western blot analysis showed a significant reduction of DPYSL2-B expression; off-target effects were screened and ruled out	Knockout reduced dendrite length, downregulated mTORC1 and cytoskeletal genes, and disrupted cholesterol biosynthesis and calcium signaling; differentially expressed genes were enriched in schizophrenia-associated loci, with alterations in protein secretion and immune pathways	The study used a single hiPSC line, with potential bypass mechanisms leading to residual CRMP2-B protein; validation is needed in diverse genetic backgrounds; long-term neurodevelopmental effects were not assessed	[113]
Investigate the role of mitochondrial dysfunction in AD-like pathology using *PITRM1*-knockout hiPSC-derived neurons and cerebral organoids	CRISPR/Cas9 was used to knock out *PITRM1* in hiPSCs by targeting exon 3 and exon 4 with specific sgRNAs	PITRM1, a mitochondrial peptidase involved in proteostasis and Aβ degradation	Mitochondrial function was assessed via Seahorse metabolic flux analysis, calcium imaging, scRNA sequencing, Western blot, and ELISA	*PITRM1* deficiency induced mitochondrial stress, increased APP and Aβ levels, and led to tau hyperphosphorylation and neuronal death in cerebral organoids, mimicking AD pathology	Organoids lacked microglia and vasculature, limiting the full replication of in vivo neuroinflammation; further validation in animal models is needed	[114]
Generation of a homozygous *ABCA7*-knockout human iPSC line using CRISPR/Cas9	Knockout of *ABCA7* gene via CRISPR/Cas9 targeting exon 5	*ABCA7* gene (exon 5, involved in lipid transport and AD risk)	Western blotting, RT-qPCR, immunocytochemistry, embryoid body differentiation, karyotyping	Generated and validated *ABCA7*^−/−^ iPSC line with normal pluripotency and neuronal differentiation capacity	Potential off-target effects minimized, but functional consequences of knockout require further validation	[115]
To develop a CRISPR-engineered human embryonic stem cell model for FTD by introducing the *CHMP2B* mutation and studying its effects on astrocyte and neuronal function	CRISPR/Cas9 genome editing using sgRNA targeting the *CHMP2B* intron 5 mutation; HDR was used with ssODN	*CHMP2B* gene, intron 5 mutation, associated with FTD	Sanger sequencing confirmed successful editing and heterozygosity.; functional characterization of neurons and astrocytes was performed via immunocytochemistry, electrophysiology, calcium imaging, and network analysis	The model exhibited reduced CHMP2B protein levels in neurons and astrocytes, impaired endolysosomal function in astrocytes, and increased glutamate uptake and calcium signaling; while individual neuronal electrophysiology remained unchanged, neuronal networks showed hyperactivity and increased synchronization, indicating altered functional connectivity	The model lacks inhibitory neurons, requires validation in patient-derived cells, does not assess long-term neurodegeneration, and shows unexpectedly low CHMP2B levels, and requires further investigation	[116]
Develop a drug-screening platform for AD using iPSC-derived cerebral organoids and CRISPR/Cas9 gene editing	CRISPR/Cas9 was used to create isogenic iPSC lines carrying the APOE ε4 variant	APOE ε4, a major genetic risk factor for sporadic AD	High-content screening, RNA-seq, mathematical modeling, and network perturbation analysis	Cerebral organoids carrying APOE ε4 showed increased Aβ and tau accumulation, altered calcium signaling, and gene expression patterns resembling AD; the platform enabled testing of FDA-approved drugs for potential repurposing	The model lacks microglia and blood vessels, requiring further validation in co-culture or in vivo models	[117]
To investigate the impact of APOE4 on human brain cell types using iPSCs and to assess whether converting APOE4 to APOE3 can reduce AD-related pathologies	CRISPR/Cas9 was used to introduce the Cys112→Arg112 substitution to generate APOE4 from APOE3 and vice versa	*APOE* gene	Editing was confirmed by Sanger sequencing, whole exome sequencing (no off-targets), and karyotyping (no abnormalities); iPSCs retained pluripotency; cell identity was validated by RNA-seq and immunostaining for neuron, astrocyte, and microglia markers	APOE4 induced AD-like changes in iPSC-derived neurons, astrocytes, and microglia, including increased Aβ, tau, and synaptic activity; APOE4-to-APOE3 conversion reversed most pathological features	In vitro iPSC models cannot fully capture the complexity of AD; APOE4-to-APOE3 conversion did not significantly reduce Aβ42 or early endosomes in sporadic AD neurons, suggesting other contributing factors; effects of the protective APOE2 allele remain poorly understood	[118]
Develop a precise and efficient method for manipulating gene dosage in human iPSCs using CRISPR/Cas9 nickases	Paired Cas9 nickases were used to create monoallelic, biallelic, or triallelic knockouts of *APP* in AD patient-derived iPSCs	*APP* gene, involved in Aβ production and tau hyperphosphorylation, a key factor in AD pathology	Genomic sequencing, immunocytochemistry, Western blot, RNA-seq, and transcriptomic analysis	*APP* gene dosage directly influenced Aβ secretion and tau hyperphosphorylation in cortical neurons derived from edited iPSCs; correction of APP copy number restored normal neuronal function and reduced apoptotic pathways	Further validation in co-culture and in vivo models is needed to confirm the therapeutic potential of gene dosage manipulation in AD	[119]
Develop an optimized toolkit for functional genomics in iPSC-derived microglia, integrating CRISPR-based gene regulation and multi-omic profiling	A drug-inducible CRISPR ON/OFF system (CRISPRi) using dCas9 for gene activation/repression without inducing DNA double-strand breaks	Multiple microglia-associated genes, including AD-linked genes such as SORL1, have been investigated for their role in neurodegenerative diseases	Extensive validation using scRNA-Seq, ATAC-Seq for chromatin accessibility, proteomics, and cytokine profiling	The optimized protocol efficiently generates microglia-like cells in 20 days, closely resembling primary human microglia in transcriptomic, epigenetic, and proteomic profiles; CRISPR ON/OFF system enables controlled gene modulation, allowing functional studies of disease-associated variants	Further in vivo validation is required; iPSC-derived microglia cultures may not fully replicate in vivo microglial responses, and additional testing in co-culture or organoid models is needed	[120]
Application of CRISPR/Cas9 for modeling neurological disorders in hiPSCs	Knockout and knock-in approaches targeting disease-associated genes	Neurological disorder-associated genes (e.g., Parkinson’s, AD, ALS-related genes)	Western blotting, qPCR, immunostaining, functional assays	CRISPR/Cas9 enables precise genetic modifications for studying neural differentiation and disease modeling	Potential off-target effects and variability in differentiation efficiency	[121]
Generation of a *DAPK1* knockout (conditional ready) human embryonic stem cell line using CRISPR/Cas9	Knockout first (conditional ready) with FRT-flanked SA-stop codon-polyA cassette	*DAPK1* gene (9q21.33, involved in apoptosis, autophagy, and inflammation)	Western blotting, qRT-PCR, IF staining, flow cytometry, karyotyping	Successfully generated a conditional-ready *DAPK1* knockout hESC line as a valuable model for studying DAPK1 in human development and diseases	Potential off-target effects were analyzed but functional implications require further studies	[122]
Generation of a miR-26b stem-loop knockout hiPSC line using CRISPR/Cas9	Knockout of miR-26b stem-loop using sgRNAs flanking the target sequence	*MIR26B* gene (2q.35, microRNA involved in gene expression regulation)	qRT-PCR, flow cytometry, immunohistochemistry, SNP analysis	Successfully generated miR-26b knockout hiPSC line with maintained pluripotency and differentiation capacity	Further functional validation is needed to assess the impact on downstream pathways	[123]
Generation and validation of *APOE* knockout human iPSC-derived cerebral organoids using CRISPR/Cas9	Knockout of *APOE* gene using CRISPR/Cas9 with gRNA pairs targeting exon 3	*APOE* gene (exon 3, conserved among all four primary transcripts)	RT-qPCR, Western blotting, immunostaining, karyotyping, single-cell clone isolation	Successfully generated APOE^−/−^ iPSC lines and validated their differentiation into cerebral organoids	Lack of vascularization and immune components in organoids may limit full physiological relevance	[124]
To identify novel atypical subgroups in amyloid-positive patients using integrated multi-omics and network modeling, aiming to uncover key AD drivers and support precision medicine strategies beyond the single-disease view of AD	CRISPR/Cas9 was used to generate isogenic APOE ε4/ε4 lines from parental APOE ε3/ε3 lines	*APOE* gene	Findings were validated using PBMCs, iPSC-derived brain organoids with microglia, and public brain transcriptome data; key drivers were confirmed in additional cohorts, including an independent Chinese AD cohort using MOFA+	Multi-omics analysis revealed novel AD subtypes linked to brain changes and driven by autophagy, immune, and lipid pathways; key genes included ABCA7, and PI3K; autophagy was dysregulated peripherally (mTOR-independent) and centrally (mTOR-dependent), especially in APOE ε4/ε4 organoids	More drug testing and long-term studies are needed; findings require validation in independent groups; due to analytical constraints, the 2-year follow-up was short, and autophagy may not be the sole clustering driver	[125]
Functionally characterize the AD-associated SNP rs148726219 using a human iPSC-based neuronal model	CRISPR/Cas9 genome editing was used to introduce heterozygous and homozygous alleles of rs148726219 in iPSCs, followed by differentiation into induced excitatory neurons	rs148726219 SNP, located in an overlapping intron of *FOSB* and *ERCC1* genes, associated with AD risk	Whole-genome sequencing and RNA-seq confirmed the edited genotypes; functional assays included calcium imaging and transcriptomic analysis.	The SNP had no impact on neuronal differentiation but led to genotype-specific transcriptional changes in mature neurons, particularly in synaptogenesis and calcium signaling pathways; homozygous edited neurons showed enhanced circuit maturation and altered unfolded protein response	Further studies are needed to explore the functional impact in additional neuronal subtypes and in co-culture models including glial cells	[126]
Generate an iPSC line carrying the AD risk variant p.S1038C in the *TTC3* gene using CRISPR/Cas9 genome editing	CRISPR/Cas9 was used to introduce the *TTC3* C>G (p.S1038C) mutation in iPSCs derived from a neurologically normal donor	*TTC3* gene, associated with late-onset AD, is involved in neuronal development and stress response	Sanger sequencing confirmed the homozygous mutation; pluripotency markers (NANOG, OCT4, SOX2) were validated using immunocytochemistry and qRT-PCR	The edited iPSC line maintained pluripotency, genomic stability, and differentiation potential, providing a resource for studying TTC3’s role in AD pathology	Further differentiation into neuronal lineages and functional assays are needed to assess the mutation’s impact on neurodegeneration	[127]
Identify neuroprotective genes and therapeutic targets for neurodegenerative diseases using a CRISPR/Cas9 genetic screen in iPSC-derived cortical neurons	Loss-of-function CRISPR/Cas9 screen using a human druggable genome sgRNA library in iPSC-derived cortical neurons	Multiple genes were identified as neuroprotective against tunicamycin-induced ER stress and neuronal death, including KAT2B	Hits validated in an arrayed format, followed by RNA-seq, proteomics, and mass spectrometry for transcriptional and protein-level analysis	The study identified 13 neuroprotective genes, including KAT2B. Pharmacological inhibition of KAT2B with L-Moses attenuated ER stress, CHOP activation, and neuronal cell death in cortical and dopaminergic neurons	Further validation in co-culture and in vivo models is needed to assess functional effects and therapeutic potential	[25]
Investigation of the impact of APOE genotype on AD pathology using CRISPR-edited isogenic iPSC lines	Knock-in conversion of APOE-ε4/ε4 to APOE-ε3/ε3 using HDR	*APOE* gene (regulation of cholesterol metabolism and AD susceptibility)	Western blotting, ELISA, immunocytochemistry, flow cytometry, genomic SNP analysis	Variability in cerebral organoid differentiation affects the reproducibility of AD phenotypes; APOE genotype showed minimal influence on Aβ and tau phosphorylation levels	High variability in organoid composition across cell lines and differentiation batches	[130]
To assess whether ABE can correct the pathogenic *SERPINI1* variant and reduce neuroserpin aggregates in patient-specific FENIB models, aiming for a targeted therapy for conformational neurodegenerative diseases	An ABE-based CRISPR/Cas9 approach was used to correct the *SERPINI1* c.1175 G>A mutation	*SERPINI1* c.1175 G>A (p.G392E) mutation in exon 9, which causes toxic neuroserpin aggregation and severe FENIB symptoms	Models validated by microscopy, Western blot, and qPCR; neuronal identity and function confirmed by markers and electrophysiology; editing checked using sequencing; aggregate clearance and morphology assessed with imaging and Sholl analysis	NG-ABE8e efficiently corrected *SERPINI1* c.1175 G>A (up to 92%), cleared aggregates, and partially rescued dendritic morphology; early treatment was more effective; engineered virus-like particlesenhanced ABE delivery (up to 38.5% editing)	No aggregation observed in knock-in neurons, likely due to short culture or low endogenous expression; overexpression may mask heterozygous phenotypes; disease modeling is limited by long aggregation timelines; genome editor delivery remains challenging	[131]
To identify the causal *CLU* variant linked to AD risk/protection and uncover its role in regulating neuronal excitability via neuron–glia lipid signaling	Isogenic iPSC lines (T/T, C/C at rs1532278) and a ~200 bp deletion in the nearby open chromatin region were generated using CRISPR/Cas9 with single-stranded oligodeoxynucleotideand paired gRNAs	The main target was SNP rs1532278 (T/C) in an intronic OCR of *CLU*, with the T allele linked to AD protection; a ~200 bp OCR deletion flanking rs1532278 was also tested for regulatory effects	Editing was confirmed with Sanger sequencing and karyotyping; iPSCs maintained pluripotency and were differentiated into brain cell types with validated identity; CLU expression was assessed using qPCR, RNA-seq, and ELISA; neuronal activity, lipid transfer, astrocyte metabolism, and glutamate uptake were tested in functional assays	The protective T allele of rs1532278 increases CLU expression in neurons, enhancing excitability, dendritic growth, and synaptic activity; neuronal CLU promotes lipid transfer to astrocytes, leading to metabolic changes and reduced glutamate uptake, which supports neuronal activity; OCR deletion reduces these effects; CLU overexpression restores them	iPSC neurons lack full maturity and aging features; in vivo validation is needed. Effects in other cell types cannot be excluded; linkage disequilibrium analysis in all culture types was incomplete; neuronal CLU may also affect astrocyte metabolism; AMPK-related mechanisms need further study	[136]
Investigate the functional role of the CLU rs11136000 SNP in astrocytes and its impact on oligodendrocyte progenitor cell proliferation and myelination in an iPSC model	CRISPR/Cas9 was used to generate isogenic iPSCs carrying either the risk “C” allele or protective “T” allele of the CLU rs11136000 SNP	CLU rs11136000 SNP, a genetic variant linked to increased risk of late-onset AD	CRISPR-edited clones were validated using genomic DNA sequencing, RNA-seq, and ELISA; functional assays included cytokine stimulation, calcium imaging, and co-culture experiments with OPCs	Astrocytes carrying the “C” allele exhibited increased CLU expression, exacerbated inflammatory response, and elevated CXCL10 levels upon cytokine treatment; these changes inhibited oligodendrocyte progenitor cell proliferation and myelination, potentially contributing to white matter deficits observed in AD patients	The study focused on in vitro models, which may not fully replicate in vivo brain conditions; further validation in co-culture and animal models is required to confirm findings	[138]
Investigate the role of CX3CR1 in human microglia using CRISPR/Cas9-edited iPSC-derived microglia-like cells	CRISPR/Cas9 genome editing targeting exon 2 of CX3CR1, followed by electroporation and FACS sorting to isolate mutant clones	CX3CR1 gene, a microglia-specific chemokine receptor involved in inflammatory responses and neuroimmune signaling	Successful knockout confirmed using Sanger sequencing and Western blot; expression changes were analyzed using qPCR and cytokine profiling	CX3CR1 knockout led to increased inflammatory responses, higher secretion of pro-inflammatory cytokines, and enhanced phagocytic activity in iPSC-derived microglia-like cells	The study focused on in vitro microglia-like cells, which may not fully replicate in vivo microglial behavior; further validation in disease models is needed	[137]
To assess the effects of chronic tau depletion on Aβ-driven toxicity in human iPSC-derived cortical neurons	Alt-R CRISPR/Cas9 system with single or paired gRNAs targeting exons 1 or 4 of the MAPT gene; Cas9-gRNA RNP delivered via electroporation	MAPT gene (exon 1 or 4 knockout)	Quantitative reverse-transcription PCR, Western blot, immunocytochemistry, long-read Nanopore sequencing, immunoprecipitation-mass spectrometry (IP-MS)	Tau depletion reduced neuronal activity, protected against Aβ-induced hyperactivity, axonal transport deficits, synapse loss, and neurodegeneration; partial tau reduction was also protective	Residual non-canonical tau peptide in exon 4 knockout lines; variable phenotypic expression among isogenic lines; Aβ insults are supraphysiological in some assays	[140]
To clarify the role of specific tau isoforms in AD by developing a human iPSC-based model and to identify toxic tau subspecies as potential therapeutic targets	MAPT knockout iPSCs were made by transfecting WTC11 iPSCs line (with dox-inducible NGN2) using Cas9 and gRNAs	Two regions in exon 1 of the *MAPT* gene, which encodes the tau protein	MAPT knockout confirmed with exon 1 sequencing and loss of tau expression (qPCR, IF, Western blot, proteomics); no off-target edits; clones showed normal karyotype, pluripotency, and neuronal differentiation	Tau knockout neurons had shorter neurites and axon initial segments, but normal activity; all tau isoforms rescued these defects; shorter isoforms sorted better to axons; knockout neurons were resistant to Aβ toxicity	iPSC neurons mainly express immature tau isoforms, differing from adult brain; neuronal activity was assessed using calcium imaging, an indirect method; study focused on acute Aβ exposure; long-term effects remain unclear; re-expressed tau isoforms showed lower axonal sorting efficiency than endogenous tau	[141]
To investigate transcriptomic changes in glutamatergic forebrain neurons derived from hiPSCs carrying FAD-related *PSEN1* mutations (A79V and L150P) using CRISPR/Cas9-edited isogenic controls	Precision CRISPR/Cas9 genome editing was used to generate isogenic control lines from patient-derived hiPSCs carrying PSEN1 mutations	*PSEN1* gene (mutations A79V and L150P)	qPCR, Sanger sequencing, and RNA-seq verification for mutation presence and absence in edited lines; CRISPRroots used to assess off-targets	1111 genes (coding and non-coding) were differentially expressed; major alterations observed in extracellular matrix-related genes, calcium signaling, and mitochondrial oxidative stress; 30 of 31 circular RNAs upregulated	model lacks full cell-type diversity (e.g., glia); RNA-seq data from only one time point; findings limited to glutamatergic neurons; trans-acting RNA effects not explored	[142]
To explore how the APOE3Ch variant protects against AD using iPSC-derived brain organoids, aiming to uncover protective pathways against tau pathology and identify therapeutic targets	CRISPR/Cas9 was used to edit patient-derived iPSCs by correcting the *PSEN1* E280A mutation and introducing or removing the APOE3Ch variant	*APOE* gene (R136S Christchurch variant) and *PSEN1* gene (E280A mutation correction)	Pluripotency (immunostaining, qPCR) and normal karyotype confirmed; gene editing validated using Sanger sequencing; AD-like pathology assessed with pTau staining; cell types identified via single-cell RNA-seq; β-catenin increase validated in patient brain (immunohistochemistry); ApoE3Ch–Wnt interaction confirmed using TCF/LEF luciferase assays	APOE3Ch reduced pTau S396 and increased β-catenin in organoids, regardless of *PSEN1* status; it altered Wnt/Cadherin pathways and promoted neuronal maturation; postmortem brain confirmed nuclear β-catenin; ApoE3Ch enhanced Wnt3a signaling, unlike ApoE3 WT	Organoids reflect early development; long-term effects need mature models; Wnt validation used non-neuronal cells; off-target edits cannot be fully ruled out; scRNA-seq and tau data may be influenced by development; APOE is mainly expressed in glia; APOE3Ch findings are based on a single rare case	[143]
To study how APOE4 drives neurodegeneration in AD using iPSC-derived brain organoids, compare APOE3/3 and APOE4/4 effects, test APOE4-to-APOE3 correction, and identify APOE4-linked disease pathways	*APOE ε4/ε4* iPSCs from an AD patient were edited to ε3/ε3	*APOE* gene	Pluripotency, karyotype, and organoid structure were confirmed; AD phenotypes were validated using immunostaining, Western blot, ELISA, and qPCR for CASP-3, synaptic markers, Aβ, tau, and APOE; transcriptomic changes were assessed by RNA sequencing and gene co-expression analysis; stress granules were evaluated by G3BP staining; appropriate statistical analyses were applied	Organoids showed AD features (Aβ, tau, neurodegeneration); APOE4 increased apoptosis, tau pathology, and stress granules; APOE4 and AD had additive effects; editing APOE4 to APOE3 reduced key AD-related phenotypes	Organoids develop necrotic cores and show size variability; protocol differences may affect results; limited iPSC lines prevented full sex matching; findings are based on in vitro models; insoluble Aβ plaques not seen at 12 weeks; longer culture needed	[145]
Examine the role of APP in neuronal development and synaptic function by generating APP-null human neurons using CRISPR/Cas9	CRISPR/Cas9 genome editing was used to knock out APP in hiPSCs, followed by differentiation into neurons using a two-step protocol	*APP* gene, linked to AD, is involved in cholesterol homeostasis and synaptic function	Genomic sequencing, immunocytochemistry, electrophysiology, live-cell fluorescence imaging, and cholesterol quantification	APP-null neurons exhibited reduced neurite growth, impaired synaptogenesis, and altered synaptic vesicle dynamics; cholesterol supplementation rescued these deficits, suggesting APP’s role in cholesterol-dependent neuronal function	Further studies are needed to confirm findings in co-culture and in vivo models to assess the broader impact of APP deficiency on neuronal function	[148]
To assess if *SORL1* loss causes endosome dysfunction in AD using hiPSC-derived cells and to test if BACE inhibition can reverse these changes.	CRISPR/Cas9 was used to knock out *SORL1* in hiPSCs by targeting exon 6 to induce frameshift indels	*SORL1* gene	Editing confirmed by sequencing and Western blot; differentiation and markers validated; endosome and APP changes were analyzed using microscopy, ELISA, and Western blot; BACE inhibitor tested; statistical controls applied	*SORL1* loss caused SORLA depletion, neuron-specific endosome enlargement, impaired APP trafficking, and increased Aβ secretion; endosome changes were BACE-independent, indicating separate AD pathways converging on endosomal dysfunction	In vitro hiPSC models may not reflect full brain complexity; only neurons and microglia-like cells were studied; the focus on SORL1 loss may not apply to FAD-linked APP mutations	[149]
To identify how rare *SORL1* missense variants found in AD patients affect SorLA maturation and trafficking, cause functional loss, and increase Aβ secretion using CRISPR/Cas9-edited hiPSCs with endogenous *SORL1* expression	Missense variants were introduced into hiPSCs using CRISPR/Cas9 with gRNAs and ssODNs via homology-directed repair; a knockout line was also generated through frameshift insertion	SORL1 gene	Edits confirmed using Sanger sequencing and Western blot; hiPSCs validated for pluripotency and karyotype; SorLA maturation, trafficking, and Aβ secretion assessed by Western blot, IF, and ELISA; structural impact predicted in silico; statistical models with correction applied.	Variants R332W, S577P, and R654W impaired SorLA maturation and trafficking, causing ER retention and reduced membrane presence; R332W and R654W increased Aβ40 secretion; structural changes likely underlie these effects; a new mechanism linking maturation defects to AD risk was identified, with rarer variants showing stronger effects	Overexpression may exaggerate effects; hiPSC models lack full brain complexity and long-term aging; only neurons and microglia-like cells were studied; variants were tested in homozygous form, unlike typical patient heterozygosity; other mechanisms were not assessed for all variants; in silico predictions did not always match experimental results	[150]
To study *ABCA7* deficiency in AD using hiPSC-derived brain cell models with homozygous *ABCA7* knockout	CRISPR/Cas9 RNP complexes were nucleofected into hiPSCs to generate *ABCA7* knockout clones	Exon 4 of the *ABCA7* gene was targeted to create a homozygous knockout using a 2-base pair deletion causing a frameshift mutation	Editing confirmed with sequencing; protein loss confirmed with Western blot; knockout hiPSCs showed normal morphology, pluripotency markers, karyotype, and three germ layer differentiation; identity confirmed by STR; mycoplasma test negative	A homozygous *ABCA7* knockout hiPSC line was created, lacking ABCA7 protein but retaining pluripotency, normal karyotype, and differentiation ability; identity matched parental cells; a hemizygous line was also generated	NA	[151]
To study how *ABCA7* deficiency affects neuronal metabolism and function, focusing on lipid metabolism and mitochondrial dysfunction in iPSC-derived models related to AD risk	*Homozygous ABCA7* knockout hiPSC lines were generated using CRISPR/Cas9 with two sgRNAs	*ABCA7* gene	*ABCA7* knockout iPSCs showed normal morphology, pluripotency marker expression, and karyotype; protein loss was confirmed by Western blot in organoids; identity was verified against parental lines	*ABCA7* knockout impaired synaptic proteins, increased oxidative stress, and disrupted mitochondrial function and morphology; neuronal activity and synapse formation decreased; lipid and NAD+ precursors rescued mitochondrial and synaptic defects; mouse models showed similar findings.	Differences between human organoids and mouse models suggest varying cellular responses; future studies should explore *ABCA7* loss on mitochondria in other brain cell types and under diverse conditions, including sex differences	[152]

AAVS1: Adeno-associated virus integration site 1; ABCA7: ATP-binding cassette subfamily A member 7; ABE: adenine base editor; AD: Alzheimer’s disease; ALS: amyotrophic lateral sclerosis; ALDH1L1: Aldehyde dehydrogenase family 1, member L1; AMPK: Adenosine monophosphate-activated protein kinase; APP: amyloid precursor protein; APOE: apolipoprotein E; APOE3Ch: APOE3 Christchurch; ATAC-Seq: assay for transposase-accessible chromatin with high-throughput sequencing; Aβ: amyloid beta; BACE: beta-site APP cleaving enzyme; Cas9: CRISPR-associated protein 9; CHMP2B: charged multivesicular body protein 2B; CLU: clusterin; CRISPR: clustered regularly interspaced short palindromic repeats; CRISPRi: CRISPR interference; CX3CR1: CX3C chemokine receptor 1; CXCL10: C-X-C motif chemokine ligand 10; DAPK1: death-associated protein kinase 1; dCas9: deactivated Cas9; DPYSL2-B: dihydropyrimidinase-like 2B; Dox: doxycycline; EGFP: enhanced green fluorescent protein; ELISA: enzyme-linked immunosorbent assay; ER: endoplasmic reticulum; ERCC1: excision repair 1; FACS: fluorescence-activated cell sorting; FAD: familial Alzheimer’s disease; FDA: Food and Drug Administration; FENIB: familial encephalopathy with neuroserpin inclusion bodies; FOSB: FosB proto-oncogene; FTD: frontotemporal dementia; GFAP: glial fibrillary acidic protein; GLAST: glutamate aspartate transporter; GPI: glycosylphosphatidylinositol; gRNA: guide RNA; HDR: homology-directed repair; hESC: human embryonic stem cell; hiPSC: human induced pluripotent stem cell; IF: immunofluorescence; IP-MS: immunoprecipitation–mass spectrometry; iPSC: induced pluripotent stem cell; KAT2B: Lysine Acetyltransferase 2B; KEGG: Kyoto Encyclopedia of Genes and Genomes; MAPT: microtubule-associated protein tau; miR: microRNA; MOFA+: multi-omics factor analysis plus; mRNA: messenger RNA; MMP: Matrix metalloproteinase-24; MT5-MMP: membrane-type 5 matrix metalloproteinase; mTOR: mechanistic target of rapamycin; mTORC1: mechanistic target of rapamycin complex 1; NAD+: nicotinamide adenine dinucleotide; NGN2: neurogenin 2; OCR: open chromatin region; PBMC: peripheral blood mononuclear cell; PI3K: Phosphoinositide 3-kinase; PITRM1: presequence protease, mitochondrial; PSEN1: presenilin 1; PSEN2: presenilin 2; qPCR: quantitative polymerase chain reaction; RNA-seq: RNA sequencing; RNP: ribonucleoprotein; RPS24: ribosomal protein S24; RT-qPCR: quantitative real-time PCR; S100β: S-100 Protein Subunit Beta; scRNA: single-cell RNA; sgRNA: single guide RNA; SNP: single-nucleotide polymorphism; SORL1: sortilin-related receptor 1; ssODN: single-stranded oligodeoxynucleotide; STR: short tandem repeat; TALEN: transcription activator-like effector nuclease; TCF/LEF: T-cell factor/lymphoid enhancer-binding factor; TTC3: tetratricopeptide repeat domain 3; T7EI: T7 endonuclease I.

## Data Availability

No new data were created or analyzed in this study.
